# Differential effects of lipid composition on the thermal and functional properties of membrane associated CYP2J2

**DOI:** 10.1002/pro.70654

**Published:** 2026-06-06

**Authors:** Rajatabha Das, Henry M. Mastrion, Harrison B. Vassar, Aditi Das

**Affiliations:** ^1^ School of Chemistry and Biochemistry, College of Sciences Parker H. Petit Institute for Bioengineering and Biosciences, Georgia Institute of Technology Atlanta USA

**Keywords:** CYP2J2, endoplasmic reticulum membrane, membrane fluidity, nano‐DSF, thermal stability

## Abstract

CYP2J2 is a membrane‐bound cytochrome P450 that is expressed in cardiomyocytes, where it is known to metabolize arachidonic acid into cardioprotective epoxyeicosatrienoic acids (EETs). It consists of transmembrane domains embedded in the hydrophobic segments of the cell membrane and surrounded by various lipids. Currently, we lack a detailed understanding of the role of specific lipids in mediating the physicochemical properties of CYP2J2 and the factors that govern this phenomenon. In this study, CYP2J2 was reconstituted into nanodiscs with different lipid compositions, selected to reflect those of the endoplasmic reticulum (ER) membrane, where the enzyme is expressed. Using a combination of Nano‐Differential Scanning Fluorimetry (nano‐DSF) and UV–Visible Spectroscopy, we demonstrate that CYP2J2 undergoes a transition in its unfolding behavior between the detergent micelle and the nanodisc environment, with the first melting transition corresponding to heme perturbation. Furthermore, we show that altering the lipid environment causes shifts of up to 3–4°C and 8–9°C in the first and second melting transition temperatures, respectively, with sphingomyelin‐ and POPS (1‐palmitoyl‐2‐oleoyl‐glycero‐3‐phosphoserine) containing nanodiscs exhibiting the highest and lowest thermal stabilities, respectively. Lipid composition was found to have no effect on substrate (ebastine) binding affinities. However, NADPH‐oxidation rates showed that lipid composition directly affects CYP2J2 function in nanodiscs by altering the rate of electron transfer between the CYP and its redox partner, Cytochrome P450 Reductase (CPR). Fluorescence anisotropy measurements with DPH (1,6‐Diphenyl‐1,3,5‐hexatriene) were also used to characterize the membrane fluidity of cholesterol‐ and sphingomyelin‐containing nanodiscs. Together, the results show that lipid composition directly modulates the thermal stability and functional properties of CYP2J2 in nanodiscs and underscore the importance of the charge of the lipid headgroup and membrane fluidity in our understanding of the mechanism by which lipid composition exerts these effects.

## 1 INTRODUCTION

Structurally, P450s consist of transmembrane domains that are embedded in the hydrophobic segments of the membrane and are surrounded by various lipids in their vicinity (Neve & Ingelman‐Sundberg, 2010). These lipids participate in the formation of direct protein‐lipid interactions as well as actively regulate membrane fluidity and packing order within the membranes (Agasid & Robinson, 2021; Deol et al., 2004; Hunte & Richers, 2008; Tieleman et al., 2021). While previous studies have shown that lipids are important for other membrane proteins, the roles of specific lipids and the factors that modulate P450 properties are not fully understood. In this study, we attempted to understand the effect of the lipid environment on the thermal and functional properties of CYP2J2 in nanodiscs. CYP2J2 is abundantly found in the cardiomyocytes and is primarily known to metabolize arachidonic acid into cardio‐protective epoxyeicosatrienoic acids (EETs) and regulate cardiovascular health, inflammation, and angiogenesis, with implications in hypertension and tumor progression (Das et al., 2020).

Thermal stability is one of the most widely used indicators for overall protein stability (Selvasingh et al., 2024). Previous studies have underscored the importance of lipid type and relative amounts in modulating the thermal stability of membrane proteins. For instance, the lipid environment has been shown to exert stabilizing effects on the conformation adopted by the transmembrane helices of the Amyloid Precursor Protein (APP) by altering membrane thickness (Dominguez et al., 2016). Lipid bilayers with higher packing order generally have a greater propensity to withstand thermal stress. This has been demonstrated with lipids such as cholesterol, which increase the thermal stability of purified rhodopsin reconstituted in mixed lipid vesicles (Bennett & Mitchell, 2008). Moreover, highly stable microdomains within the ER membrane, known as Detergent‐Resistant Micelles (DRMs), which regulate several cellular processes such as signal transduction, endocytosis, and protein trafficking by serving as platforms for lipid‐protein and protein–protein interactions, are particularly enriched in cholesterol and sphingomyelin (Sferra et al., 2025). This suggests a strong correlation between lipid composition and membrane stability. A recent study using CYP3A4 in nanodiscs demonstrated the stabilizing effect of lipids with saturated fatty acyl chains, which promote tighter packing of the lipids, with DPhPC nanodiscs showing an increase of 4°C in the protein's melting temperature (Knetsch & Ubbink, 2024). In another study, the bacterial disulfide bridge‐forming protein (DsbB) was found to exhibit different thermal stabilities and unfolding patterns when reconstituted into nanodiscs of varying lipid compositions.

**FIGURE 1:**
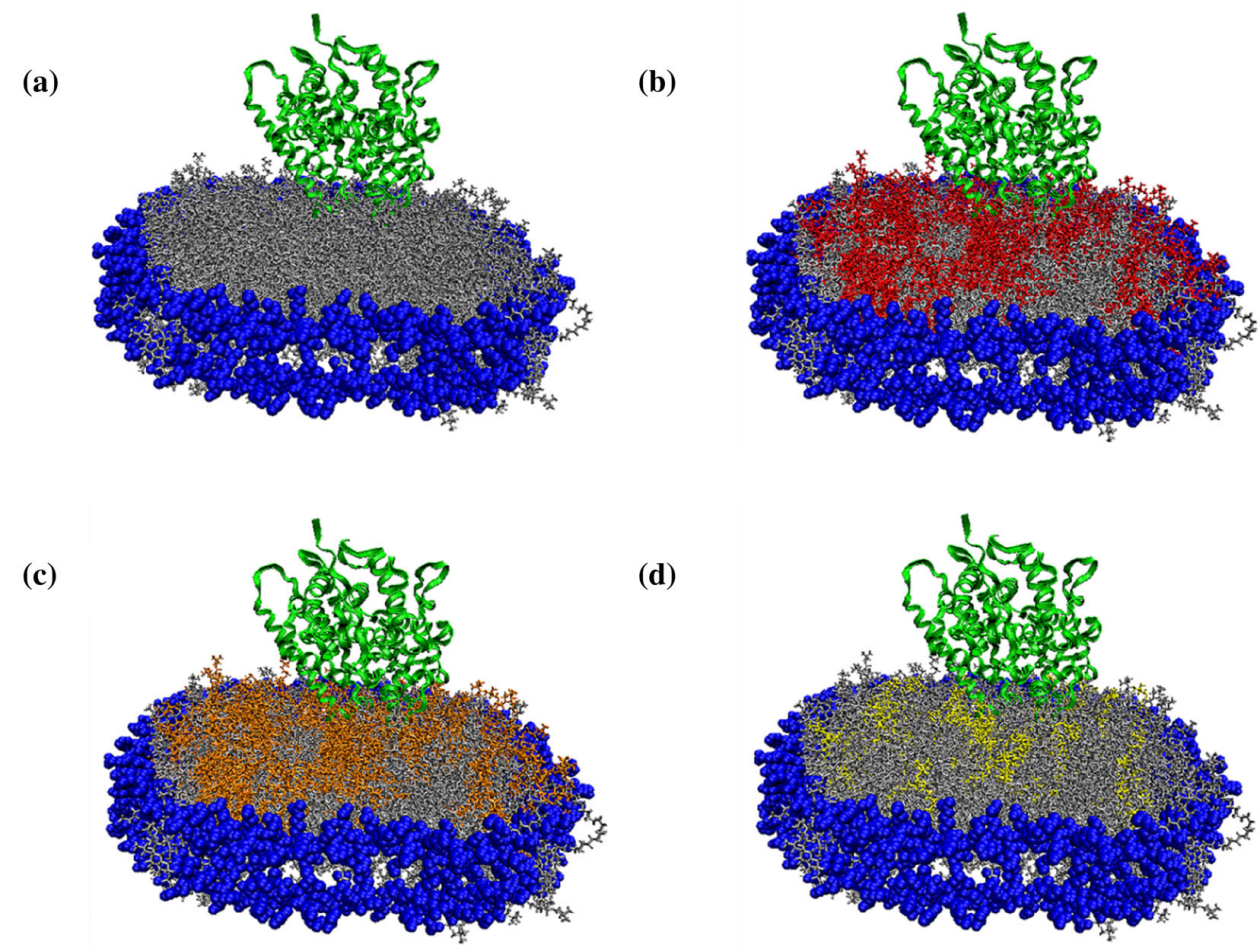
Visual molecular dynamics (VMD) schematic of CYP2J2 incorporated in (a) 100% POPC (b) 30% POPS (c) 30% POPE and (d) 10% sphingomyelin nanodiscs. In the model, blue represents the membrane scaffold protein (MSP1D1), POPC (silver), POPS (red), POPE (orange), sphingomyelin (yellow) and CYP2J2 is represented as the protein ribbon structure in green. These schematics are for illustrative purposes only.

Most biophysical studies require the protein to be stable outside of its native membrane environment. With hydrophobic segments of membrane proteins conferring hurdles of low solubility and stability, and detergent micelles having well‐documented detrimental effects on the structural and functional integrity of the protein, nanodiscs have emerged as an alternative membrane mimic for the stabilization of membrane proteins (Denisov & Sligar, 2016; Guo, 2021; Laurence et al., 2022). Nanodiscs are nanoscale lipid bilayer mimics whose size is controlled by the membrane scaffold protein (MSP) (Figure 1). In this study, we use nanodiscs as a platform for modulating lipid composition. Previously, nanodiscs have been widely used for a variety of biophysical techniques such as Differential Scanning Calorimetry (DSC), Dynamic Light Scattering (DLS), Surface Plasmon Resonance (SPR), Small‐angle X‐ray scattering (SAXS), and Solution Electron Microscopy (Solution‐EM) (Bengtsen et al., 2020; Carvalho et al., 2018; Daniilidis et al., 2022; Nakagawa et al., 2023; Reis & Moraes, 2020; Tahmasbi Rad et al., 2021; Zhou et al., 2019) (Smith et al., 2024). While they offer several advantages such as better solubility and stability, small size of the nanodisc assembly as well as homogeneity in the sample preparation, they also have several limitations, such as lacking an asymmetric bilayer, curvature in the membrane and having size limitations while incorporating larger proteins (Young, 2023). More recently, they have garnered a lot of interest due to their ability to deliver hydrophobic drugs and enzymes to various locations inside the body (Stępień et al., 2023). With the increasing interest in utilizing nanodisc technology for these delivery purposes, it is important to understand the fundamental role of lipids in mediating the stability of membrane proteins.

Lipid composition has also been shown to modulate the function of membrane proteins. Previously, titrating phospholipids containing a choline or ethanolamine head group along with cardiolipin into a previously delipidated cytochrome bc1 has been shown to lead to maximum restoration of activity (Schägger et al., 1990). Similarly, replacement of the choline head‐group on a phospholipid with a glycerol head‐group for a bacterial translocase protein resulted in restoration of activity, thereby underlining the importance of specific anionic phospholipids for the function of such proteins (van der Does et al., 2000). In another study, different lipid compositions were found to stimulate CYP1A2 activity by increasing the efficiency of the formation of the CYP1A2‐CPR complex (Brignac‐Huber et al., 2011). Moreover, lipid composition was found to directly affect the rate and pattern of ebastine hydroxylation by CYP2J2 in nanodiscs. Interestingly, the incorporation of sphingomyelin promoted the formation of Carebastine from Hydroxyebastine (primary product) through processive metabolism, suggesting that sphingomyelin might induce an active‐site conformational change in CYP2J2 that stabilizes the hydroxylated intermediate of ebastine, leading to a second round of metabolism (Huff et al., 2019). Membrane proteins such as P450s are often the focus of extensive functional and metabolic studies with various lipid and drug substrates. Therefore, knowledge and supplementation of the precise lipid environment to maximize enzyme activity become important.

Herein, we studied the effect of lipid composition on the thermal stability and function of CYP2J2 in nanodiscs. To ensure a controlled lipid environment, we reconstituted CYP2J2 in nanodiscs using representative ER membrane lipids, where the enzyme is abundantly expressed. Most studies probing the effect of lipid composition on thermal stability using DSC (McClary et al., 2016). While DSC is an established, robust method of measurement, we use Nano‐Differential Scanning Fluorimetry (Nano‐DSF) to probe the thermal stability of proteins due to the requirement of low sample volume, enabling the study of various lipid compositions in a high‐throughput manner (Selvasingh et al., 2024). To ensure that the nanodisc itself does not interfere with thermostability measurements of the target protein using nano‐DSF, we prepared the nanodiscs using a modified MSP construct, which renders it “fluorescently dark” (Selvasingh et al., 2024). We used UV–Visible spectroscopy to assess whether heme dissociation from the active site of cytochrome P450 is a likely event throughout the unfolding process. The nano‐DSF measurements identified the lipid compositions contributing to the highest and lowest thermal stability. Moreover, binding studies using UV–Visible spectroscopy were conducted to investigate the effect of the lipid environment on the enzyme's substrate‐binding capabilities. Furthermore, fluorescence anisotropy measurements were used to characterize selected lipid compositions with respect to membrane fluidity and packing order. NADPH oxidation assay results showed that lipid composition directly affects enzyme activity by altering the rate of electron transfer between CYP and its redox partner, CPR. Taken together, in this study, we investigate the role of the lipid environment in modulating membrane protein properties and delineate the factors that govern this process.

## 2 RESULTS

### 2.1 Reconstitution of CYP2J2 into nanodiscs of different lipid composition

**FIGURE 2:**
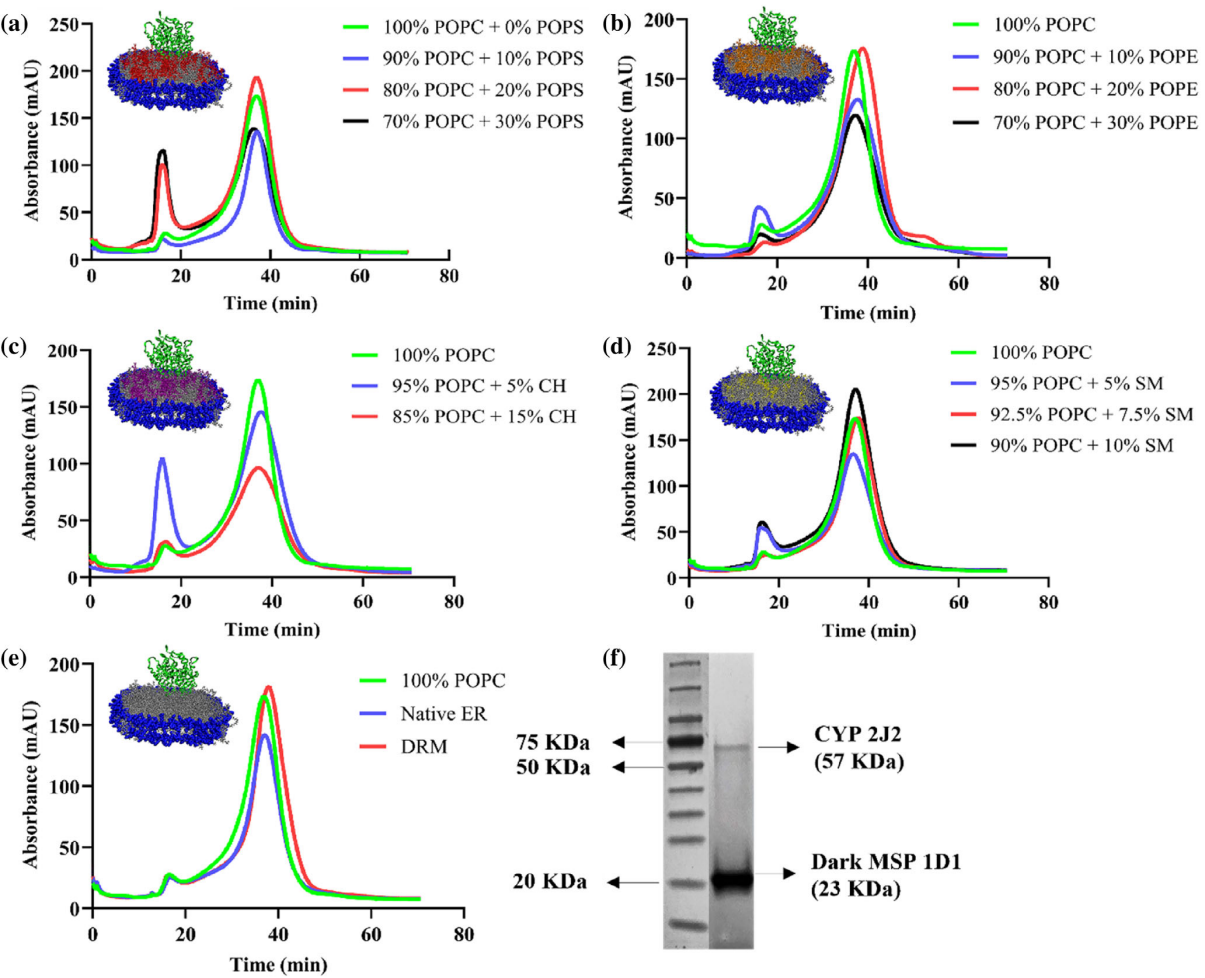
Size‐exclusion chromatograms of CYP2J2 incorporated into (a) POPC‐POPS (b) POPC‐POPE (c) POPC‐cholesterol (d) POPC‐sphingomyelin and (e) Native ER and DRM lipid composition nanodiscs. The absorbance was measured at 280 nm. The nanodiscs eluted at around ~37 min (~15 mL of retention volume using 0.4 mL/min as flow rate) in a Superdex 200 10/300 GL size exclusion column in 0.1 M potassium phosphate buffer (pH 7.4). (f) SDS‐PAGE of CYP2J2 nanodisc (100% POPC) showing two distinct bands for CYP2J2 (~57 KDa) and dark MSP1D1 (~23 KDa).

The expression of an N‐terminus‐truncated construct of CYP2J2, co‐transformed with the molecular chaperone GroEL/ES, was performed as described in the previous literature (McDougle et al., 2013). CYP2J2 was purified using Ni‐NTA affinity chromatography. Lipids used to make the nanodiscs were chosen based on the subcellular localization of CYP2J2. Since the enzyme is predominantly expressed in the endoplasmic reticulum (ER) membrane of cardiomyocytes, the reported native ER lipid composition was used as a reference point (Tomczyk & Dolinsky, 2020) (Pradas et al., 2018) (van Meer et al., 2008). We made CYP nanodiscs with the following lipids: POPC (1‐palmitoyl‐2‐oleoyl‐glycero‐3‐phosphocholine), POPS (1‐palmitoyl‐2‐oleoyl‐glycero‐3‐phosphoserine), POPE (1‐palmitoyl‐2‐oleoyl‐glycero‐3‐phosphoethanolamine), cholesterol and sphingomyelin. While POPC (70%–100%) and POPE (10%–30%) were used due to their high abundance in the ER membrane, POPS (10%–30%) was used to introduce anionic lipid content and study the effect of the lipid headgroup charge on the properties of the protein. In addition, since cholesterol and sphingomyelin levels were found to be more enriched in Detergent Resistant Micelles (DRMs), which are extremely stable microdomains found within the ER membrane, these lipids were also included to probe their role in mediating stability. Moreover, the reported native ER (70% POPC, 1% POPS, 20% POPE, 5% CH and 4% SM) and DRM lipid composition (45% POPC, 2% POPS, 18% POPE, 23% CH and 12% SM) were also used to make CYP2J2 nanodiscs, as references for comparison (Brignac‐Huber et al., 2011). Although POPS constitutes only ~1% of the native ER membrane, narrow variations around this level are unlikely to produce measurable effects in reconstituted systems. Therefore, to directly assess the influence of a negatively charged lipid head group, POPS content was varied over a broader range (10%–30%), enabling clear evaluation of charge‐dependent effects on protein stability and activity (Huff et al., 2019). For POPE, a 20% composition was selected to reflect the native ER membrane. To examine how deviations from this baseline affect CYP2J2 properties, additional formulations with lower (10%) and higher (30%) POPE content were included, allowing assessment across a physiologically relevant range. For cholesterol and sphingomyelin, lipid amounts were guided by their reported abundances in the ER and detergent‐resistant membrane (DRM) fractions, which represent lower and upper compositional extremes, respectively. Accordingly, compositions were chosen to span this range. For example, sphingomyelin content was varied from near‐ER levels (5%) to intermediate levels (7.5%) to levels approaching DRM levels (10%) (Brignac‐Huber et al., 2011). This strategy enabled us to investigate how increasing membrane order and heterogeneity influence the behavior of CYP2J2. All the nanodiscs were prepared with a two‐lipid system with POPC as the base lipid since it is the most abundant lipid in the ER membrane. A schematic showing CYP2J2 incorporated into nanodiscs of different lipid composition is shown in Figure 1. The 14 different lipid compositions used to make the CYP2J2 NDs have been listed in Table 1. CYP2J2 was assembled into homogeneous, uniform nanodiscs by size‐exclusion chromatography. The nanodiscs eluted at ~37 min (15 mL retention volume at 0.4 mL/min). The corresponding chromatograms of the nanodiscs with different lipid compositions are shown in Figure 2a–e. The successful incorporation of CYP2J2 into nanodiscs was verified by SDS‐PAGE (Figure 2f), which shows two distinct bands corresponding to CYP2J2 (~57 kDa) and dark MSP1D1 (~23 kDa).

**TABLE 1:** Lipid compositions used for making all varieties of CYP2J2 NDs.

Nanodisc	POPC%	POPS%	POPE%	CH%	SM%
1	100	0	0	0	0
2	90	10	0	0	0
3	80	20	0	0	0
4	70	30	0	0	0
5	90	0	10	0	0
6	80	0	20	0	0
7	70	0	30	0	0
8	85	0	0	15	0
9	75	0	0	25	0
10	95	0	0	0	5
11	92.5	0	0	0	7.5
12	90	0	0	0	10
Native ER	70	1	20	5	4
DRM	45	2	18	23	12

### 2.2 Difference in thermal unfolding of CYP2J2 in detergent versus nanodisc

As shown in Figure S4, Nano‐DSF relies on the red shift in the fluorescence emission spectra (330 to 350 nm) upon exposure of buried tryptophan residues to the polar environment, as the protein unfolds (Kim et al., 2021). The MSP used to make the nanodiscs convolutes fluorescence measurements since it also contains tryptophan residues. While previous studies have used empty nanodiscs as the control, we have used modified MSP constructs, where tryptophan residues are mutated to phenylalanine to render them fluorescently dark and named them “dark MSP” (Knetsch & Ubbink, 2024) (Selvasingh et al., 2024) (McLean et al., 2020). This enabled us to study the unfolding of the protein using nano‐DSF without any contribution to the fluorescence signal from the tryptophan residues in the MSP. Figure S2 shows the negligible fluorescence contribution from our dark MSP construct, while the other MSP construct shows significant fluorescence.

**FIGURE 3:**
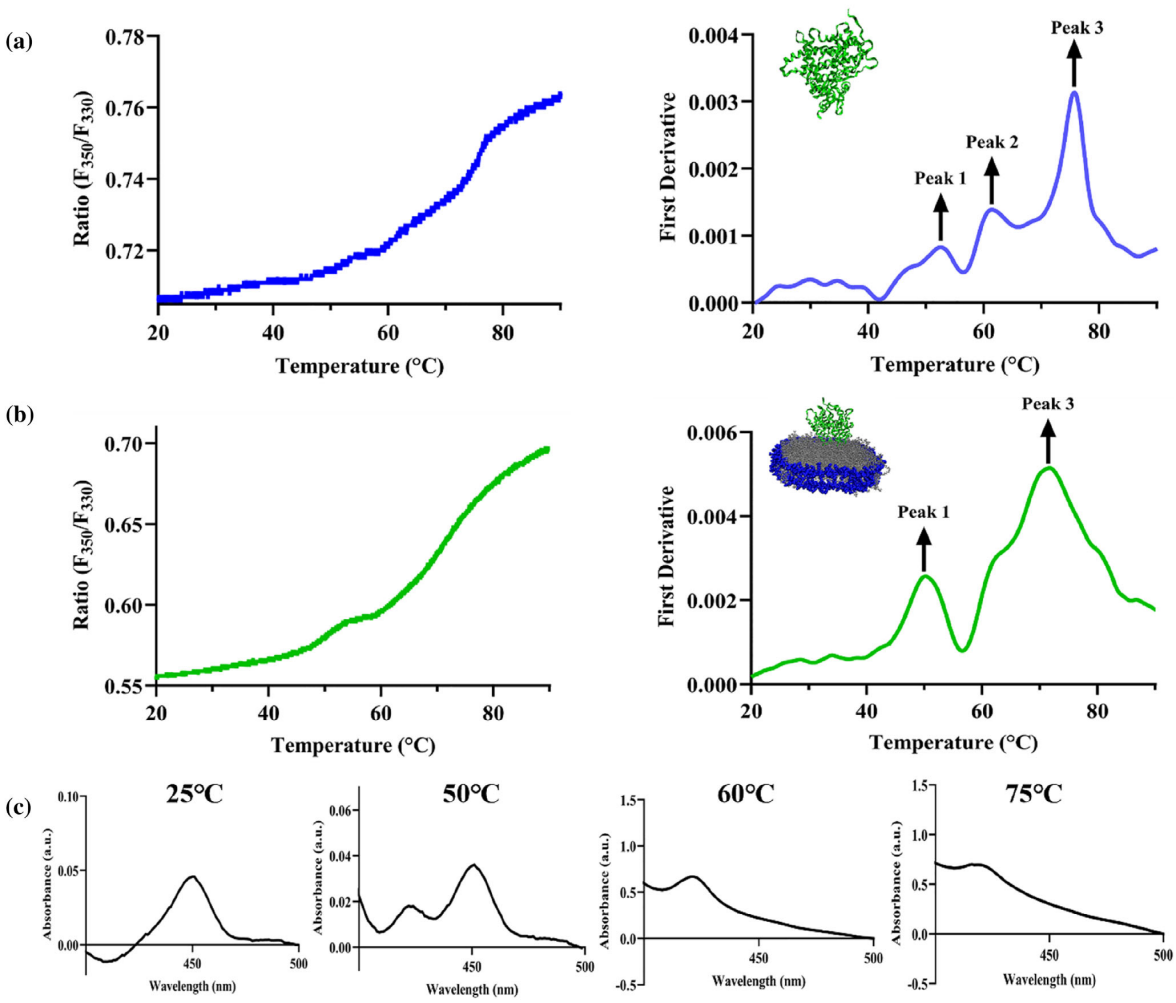
Differential unfolding behavior of CYP2J2 in detergent (cholate) versus nanodisc. Ratio of fluorescence intensity at 350 and 330 nm (F_350_/F_330_) and first derivative plotted against a thermal ramp from 20 to 90**°**C for CYP2J2 in (a) detergent micelles (cholate) and (b) 90% POPC‐10% POPS nanodisc. Peak 1 (~50**°**C) and Peak 3 (~75**°**C) remain conserved while Peak 2 (~60**°**C) becomes less prominent and merges with Peak 3 when CYP2J2 is incorporated into nanodiscs. Average of triplicates has been plotted for both (a) and (b). (c) Ferrous–CO binding assay for CYP2J2 native ER ND incubated at 25, 50, 60, and 75**°**C for 10 min showing perturbation of the heme environment on thermal unfolding. Data shows complete conversion of P450 to P420 and inactivation of CYP2J2 on heating beyond first melting transition temperature. The appearance of the P420 peak and disappearance of the P450 peak points to possible ligand switching from cysteine to histidine or thiolate protonation.

NanoDSF measurements of CYP2J2 in detergent micelles reveal three peaks in the first‐derivative plot in Figure 3a. The detergent used for these experiments is 0.1% v/v cholate. While the fluorescence ratio (F350/F330) reports gradual temperature‐dependent changes in the local environment of aromatic tryptophan residues, the first derivative highlights the temperature at which this change is most rapid, corresponding to the inflection point of the ratio curve and the apparent melting temperature. Each peak in the first‐derivative plot corresponds to a distinct transition in the unfolding process. The three transitions occur around 50, 60, and 75°C. The thermal unfolding of P450 enzymes has been a major focus of many studies. The major melting transition of CYP3A4 in POPC NDs, studied using DSC, occurred around 50°C and was composed of several smaller transitions at 46, 53, and 56°C (McClary et al., 2016). Similarly, in another previous study, the thermal unfolding process was shown to be primarily composed of two major transitions. The first one, occurring at around 50°C, is not a sharp peak in the DSC endotherm and can be broken down into several smaller transitions at 40, 49.5, 54, and 60°C (Anzenbacher et al., 1982). This is consistent with our observation that the first two transitions of CYP2J2 in detergent micelles occur at 50 and 60**°**C. However, while they report their second major transition occurring around 90°C, we observe the second transition at around 75**°**C for CYP2J2 (Anzenbacher et al., 1982). In the previous study, a collection of P450s isolated from liver microsomes of phenobarbital‐treated rats was used for the DSC experiments. The broad transition around 50**°**C is usually attributed to the unfolding of different regions of the enzyme with varying stabilities. Interestingly, the thermal unfolding behavior of CYP2J2 incorporated into a nanodisc differs from that of CYP2J2 in detergent micelles. In CYP2J2 ND unfolding studies using NanoDSF, two distinct, dominant peaks are observed in the first‐derivative plot, corresponding to two major unfolding transitions. Moreover, as shown in Figure 3b, when comparing the melting transitions of CYP2J2 inside and outside the nanodisc, it becomes evident that the middle transition (~60**°**C) becomes less prominent when the protein is in the bilayer environment, while the other two transitions remain largely conserved along with their relative intensities. This underscores the lipid bilayer's ability to influence the protein's unfolding behavior.

### 2.3 The first melting transition corresponds to heme dissociation

Although there have been studies primarily focused on the unfolding of P450 enzymes, the physical and structural significance of these different unfolding transitions remains relatively underexplored. For heme‐containing enzymes such as the P450 family, it is hypothesized that disturbance of the heme environment is the first step in protein unfolding. Denaturation of CYP2B1 in the presence of GdHCl (Guanidium hydrochloride) and urea has been studied previously. It was reported that the denaturation of CYPs is a two‐step process: an undefined change in the active site leading to inactivation, followed by the disturbance of heme binding leading to a change in the absorbance of the Soret peak at 417 nm or the loss of the absorbance peak in the reduced carbon monoxide difference spectrum at 450 nm (Yu et al., 1995). Heme perturbation was studied using the absorbance at 360 nm, which has often been attributed to that of dissociated heme remaining loosely attached (non‐covalently and/or out of the heme pocket) to the protein matrix (Kundu et al., 2015). To monitor this, purified CYP2J2 in nanodiscs made using the reported native ER lipid composition was incubated at discrete temperatures ranging from 30 to 60**°**C for 10 min. The nanodisc sample was allowed to equilibrate at each temperature, and the ratio of absorbance at 360 nm to absorbance at 417 nm was plotted as a function of temperature. The A_360_/A_417_ ratio was used as a measure of heme perturbation in P450s as temperature increased. We would expect a decrease in the absorbance at 417 nm (intact heme) and a rise in the absorbance at 360 nm (perturbed heme) with increasing temperature. A similar methodology has previously been applied to study thermal unfolding of CYP2C8 (Sun et al., 2010). The extent of the perturbation does not change significantly until a threshold temperature of 40°C, then increases gradually until 47.5°C, followed by a sharp increase around 50°C. Figure S5 shows that A_360_/A_417_ increases with temperature, supporting the hypothesis that the heme environment is significantly perturbed upon thermal denaturation or stress.

An alternative way to study heme perturbation resulting from thermal denaturation is to perform CO‐binding assays with CYP2J2 NDs at various temperatures. This has previously been done to investigate the thermostability of polymorphic variation of CYP3A4 and CYP2C9 (Arendse & Blackburn, 2018). To further probe this, we performed CO‐binding assays with CYP2J2 native ER NDs incubated at four different temperatures: 25°C (Room temperature), 50°C (before the first melting temperature), 60°C (after the first melting temperature), and 75°C (Second melting temperature). As shown in Figure 3c, we used ferrous–CO difference spectra to correlate changes in heme ligation with the thermal unfolding transitions observed by nanoDSF. At 25°C, the spectrum is dominated by the characteristic P450 peak at 450 nm with a negative feature near 420 nm, consistent with a fully folded, thiolate‐ligated heme environment. At 50°C, corresponding to temperatures just below the first thermal transition detected by nanoDSF, the 450 nm signal decreases while a pronounced positive P420 peak emerges. Using the empirical constants and readings from the final CO‐binding spectra, the relative percentages of P450 and P420 were found to be 70.8% and 29.2%, indicating partial conversion to the inactive P420 species in a subpopulation of protein molecules. Upon heating beyond the first melting transition, the P450 species is no longer detectable; at both 60 and 75°C, only the P420 form is observed, indicating 100% conversion of P450 to P420.

The characteristic absorption band at 450 nm observed in ferrous–CO complexes is a defining spectroscopic feature of cytochrome P450 enzymes and arises from coordination of the heme iron by a cysteine thiolate ligand. In contrast, most other heme proteins employ neutral axial ligands, such as histidine. Disruption of the native P450 fold by harsh chemical or physical treatments commonly leads to the appearance of a reduced‐CO absorption band near 420 nm, corresponding to formation of the inactive P420 species, which reflects alteration of the proximal heme ligation and loss of catalytic competence (Lipscomb, 1980; Martinis, Blanke, et al., 1996) (Luthra et al., 2011). The appearance of a P420 peak has been attributed to multiple possible events. More specifically, the P420 peak at 420 nm has been rationalized through protonation of the cysteine thiolate ligand, complete dissociation of the cysteine ligand, or replacement of the cysteine ligand by histidine (Gable et al., 2022; Sun et al., 2013). The complete loss of the P450 signal coincident with crossing the first unfolding transition demonstrates that disruption of cysteine thiolate coordination and stabilization of the P420 state accompany early stages of thermal denaturation, preceding or occurring alongside global structural unfolding.

### 2.4 Lipid composition affects thermal stability of CYP2J2 in nanodisc

**FIGURE 4:**
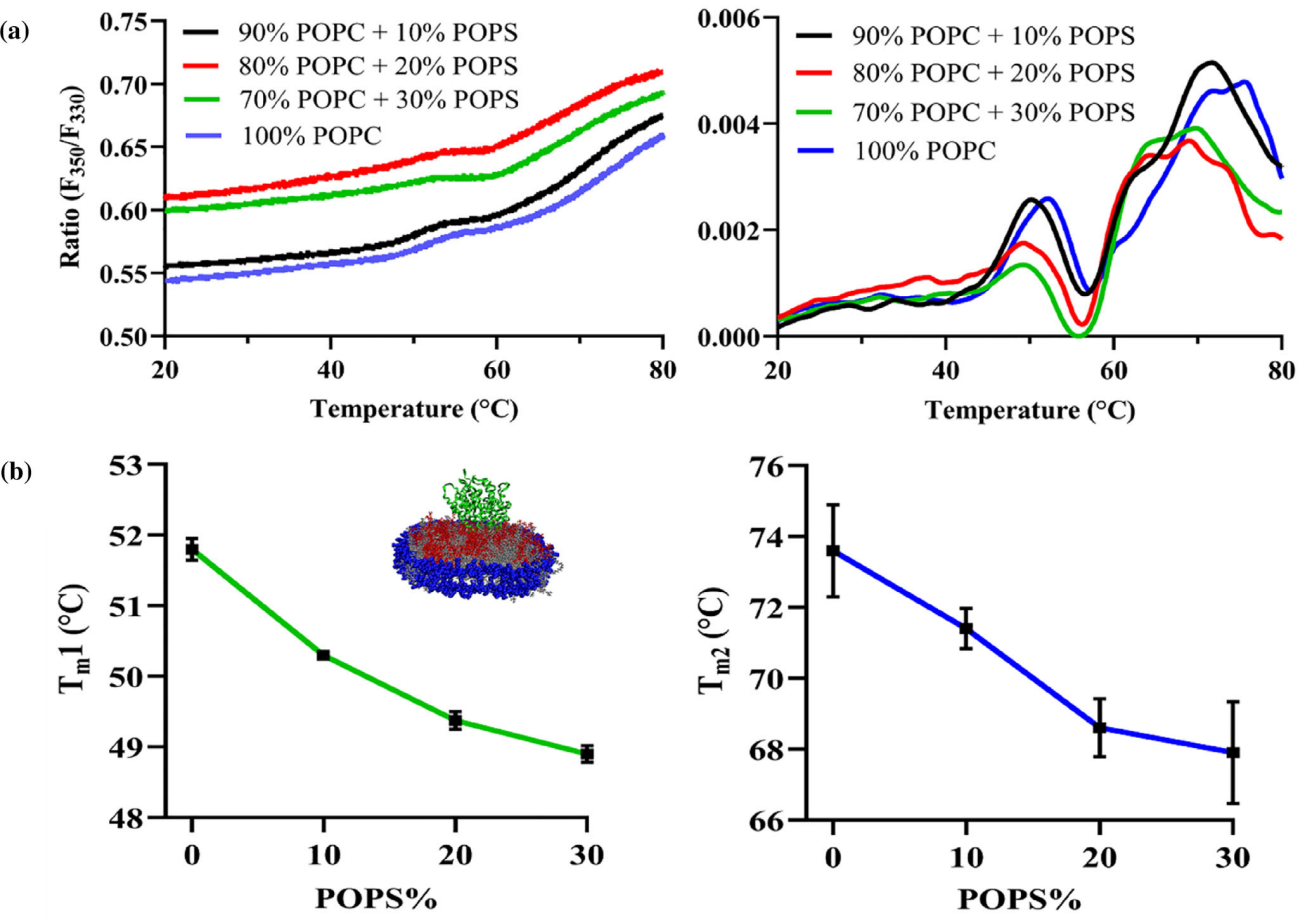
Effect of addition of a negatively charged lipid POPS to the lipid composition on the thermostability of CYP2J2 in nanodisc. (a) Ratio of fluorescence intensity at 350 and 330 nm (F_350_/F_330_) and first derivative plotted against a thermal ramp from 20 to 90**°**C for CYP2J2 in 0, 10, 20 and 30% POPS nanodiscs. Average of triplicates has been plotted for both plots. (b) Variation of two melting transition temperatures (T_m_1 and T_m_2) against the POPS% in nanodisc composition showing CYP2J2 becomes thermally unstable with increasing POPS content. Average of triplicates is plotted with SEM as error bars.

**FIGURE 5:**
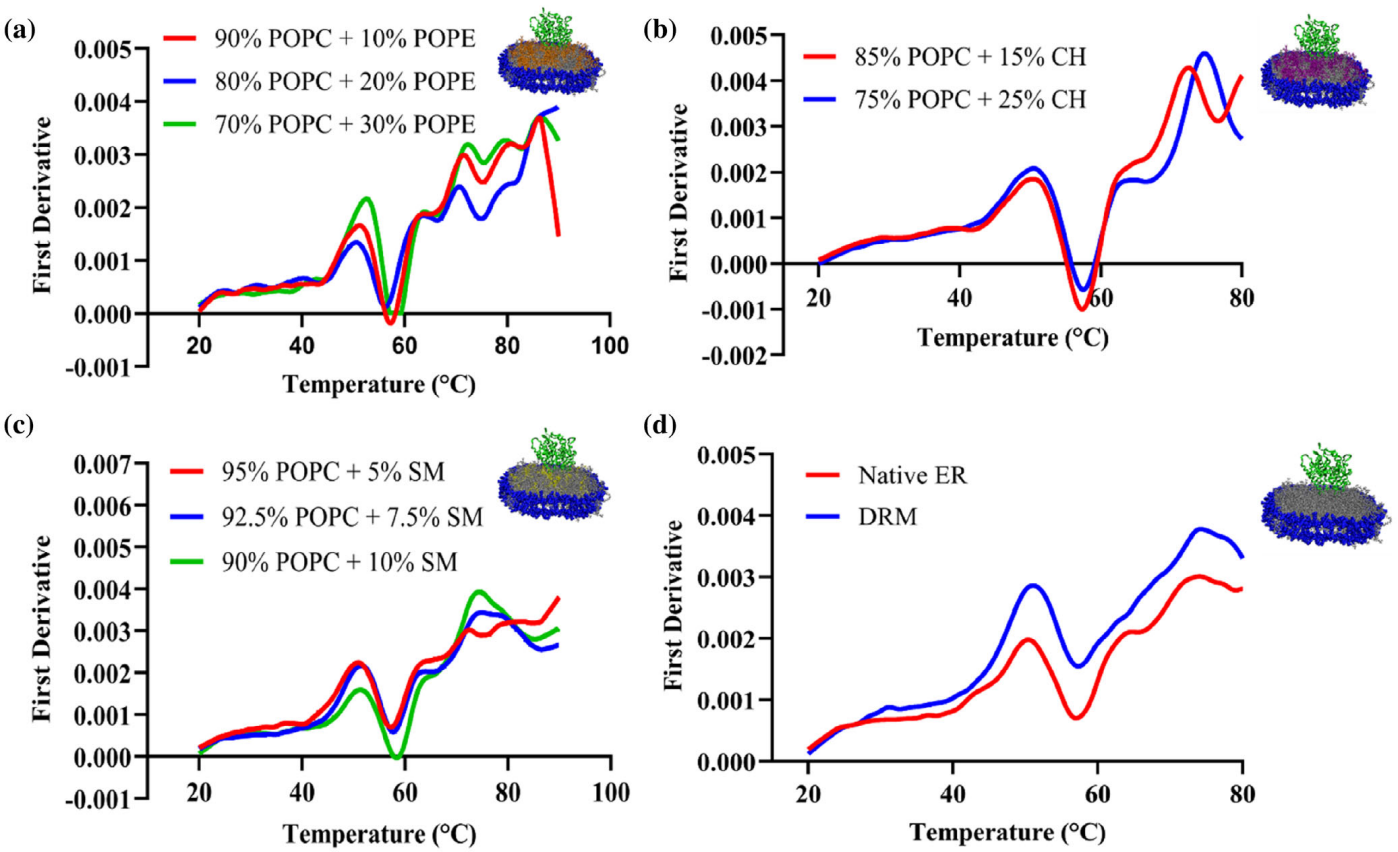
Effect of lipid composition on thermal stability of CYP2J2 in nanodisc. First derivative plotted against a thermal ramp from 20 to 90**°**C for CYP2J2 in (a) POPC‐POPE, (b) POPC‐cholesterol, (c) POPC‐sphingomyelin, (d) Native ER and DRM nanodiscs. Average of triplicates has been plotted for all the plots.

CYP2J2 incorporated into 14 different mixed‐lipid nanodiscs was subjected to thermal unfolding studies using nano‐DSF. Additionally, using peak analysis of the first‐derivative plots, the variation in the melting temperatures of the two unfolding transitions of CYP2J2 in nanodiscs was analyzed as a function of lipid composition. As shown in Figure 4a, for POPC‐POPS nanodiscs, we observed a shift of the two peaks in the first derivative plot toward the left with increasing POPS%. To study this further, we plotted the variation in the two T_m_ values against the POPS concentration in the nanodisc. As shown in Figure 4b, both T_m_1 and T_m_2 decrease as POPS% increases. Upon increasing the POPS% from 0% to 30%, we observed a change of roughly 3°C in T_m_1 and 6°C in T_m_2. This indicates that the protein becomes destabilized upon the introduction of anionic phospholipids in the lipid bilayer. Interestingly, as shown in Figure 4, for POPS, POPE, and CH‐containing compositions, the second transition peak (around 75°C) in the first derivative plot becomes less sharp and is broken down into smaller peaks. In these cases, the local maxima in the 70–75**°**C range were taken to be T_m_2, since the second melting transition temperature was observed in this temperature range for other lipid compositions. The T_m_2 for these lipid compositions, therefore, needs to be interpreted as an apparent or broadened transition rather than a discrete melting event. The transition to a broader overall peak or to smaller composite peaks, however, is not observed across all lipid compositions, indicating that this effect depends on lipid composition. It has been previously suggested that the peaks in the nanoDSF first‐derivative plots correspond to different regions or domains of the enzyme denaturation with different thermal stabilities (Gao et al., 2020). It is possible that protein‐lipid interactions arising from dynamic, low‐affinity contacts between residues in different regions of the enzyme with bulk and boundary lipids lead to differential unfolding behavior. Alternatively, the individual peaks in the first‐derivative plot can correspond to distinct protein conformations, and the redistribution of the peak at the second melting transition temperature into smaller peaks for some of the NDs can indicate a potential shift in the conformational equilibrium (Lisina et al., 2023). More specifically, POPE has been shown to alter conformational states of proteins. For instance, PE has been shown to be required at the late step of the conformational maturation of the polytopic membrane protein lactose permease (Bogdanov et al., 2002; Bogdanov & Dowhan, 1998). In another study, the addition of PE lipids in LmrP reconstituted in PC containing liposomes resulted in a reduction in the energy barrier between conformational states, leading to greater conformational flexibility and switching between inward and outward‐facing states (Hakizimana et al., 2008). Similarly, studies of the p24 transmembrane domain reconstituted into SM containing membranes showed altered transmembrane helix orientation, topology, and dynamics, demonstrating that SM can directly modulate protein conformational states (Aisenbrey et al., 2019). POPS has also been shown to affect the conformational dynamics of GPCRs and to bind at specific sites, modulating gating conformations of Kir channels (Dijkman et al., 2020; Duncan et al., 2020; Thakur et al., 2023). The presence of these lipids might be responsible for the protein populating different conformational states and leading to multiple peaks near the second melting temperature in the first derivative plot. Table 2 shows the variation of the two melting temperatures across 14 different lipid compositions. As shown in Figure 5a–b, upon analyzing trends in lipid composition, we observe a moderate decrease in both melting transition temperatures with increasing POPE and CH content in the nanodisc. However, for POPC‐SM NDs (Figure 5c), increasing SM content increases T_m_1 and T_m_2. Upon comparison, we see a difference of 3–4**°**C for T_m_1 and a shift of roughly 8–9**°**C between 10% SM NDs and 30% POPS NDs for T_m_2. This is a big change in the thermal stability of CYP2J2, given that previous studies with CYP3A4 shifted the melting temperature by 3–4°C by changing the lipid composition (McClary et al., 2016) (Knetsch & Ubbink, 2024). Furthermore, this underscores the role of SM in mediating stability, since DRMs are also known to be particularly stable structures with high SM content (van Gestel et al., 2016). As is evident, lipid composition directly impacted the thermal stability of CYP2J2 in nanodiscs, with the introduction of POPS and sphingomyelin resulting in the lowest and highest thermal stabilities, respectively. All nanoDSF ratio (F350/F330) plots versus temperature for all lipid‐composition nanodiscs are shown in Figure S1.

**TABLE 2:** NanoDSF melting transition temperatures of all 14 mixed‐lipid CYP2J2 NDs. Temperatures are average of measurements in triplicates. All errors shown in the table are standard deviations. (n=3).

Nanodisc	T_m_1 (°C)	T_m_2 (°C)
100% POPC	51.70 ± 0.3	73.60 ± 0.2
10% POPS	50.33 ± 0.2	71.40 ± 0.4
20% POPS	49.35 + 0.5	68.60 ± 0.5
30% POPS	48.9 ± 0.4	67.90 ± 0.7
10% POPE	51 ± 0.3	71.40 ± 0.8
20% POPE	50.45 ± 0.9	70.40 ± 0.3
30% POPE	50.225 ± 1.0	70.23 ± 0.2
15% CH	50.26 ± 0.2	74.76 ± 0.5
25% CH	50.16 + 0.5	72.40 ± 0.3
5% SM	50.80 ± 0.4	72.32 ± 0.5
7.5% SM	51.23 ± 0.3	74.60 ± 0.6
10% SM	52.40 ± 0.2	76.56 ± 0.2
Native ER	51.03 ± 0.3	74.8 ± 0.6
DRM	50.36 ± 0.5	73.5 ± 0.2

### 2.5 Lipid composition affects the function of CYP2J2 in nanodisc by altering the rate of electron transfer between CYP‐CPR

**FIGURE 6:**
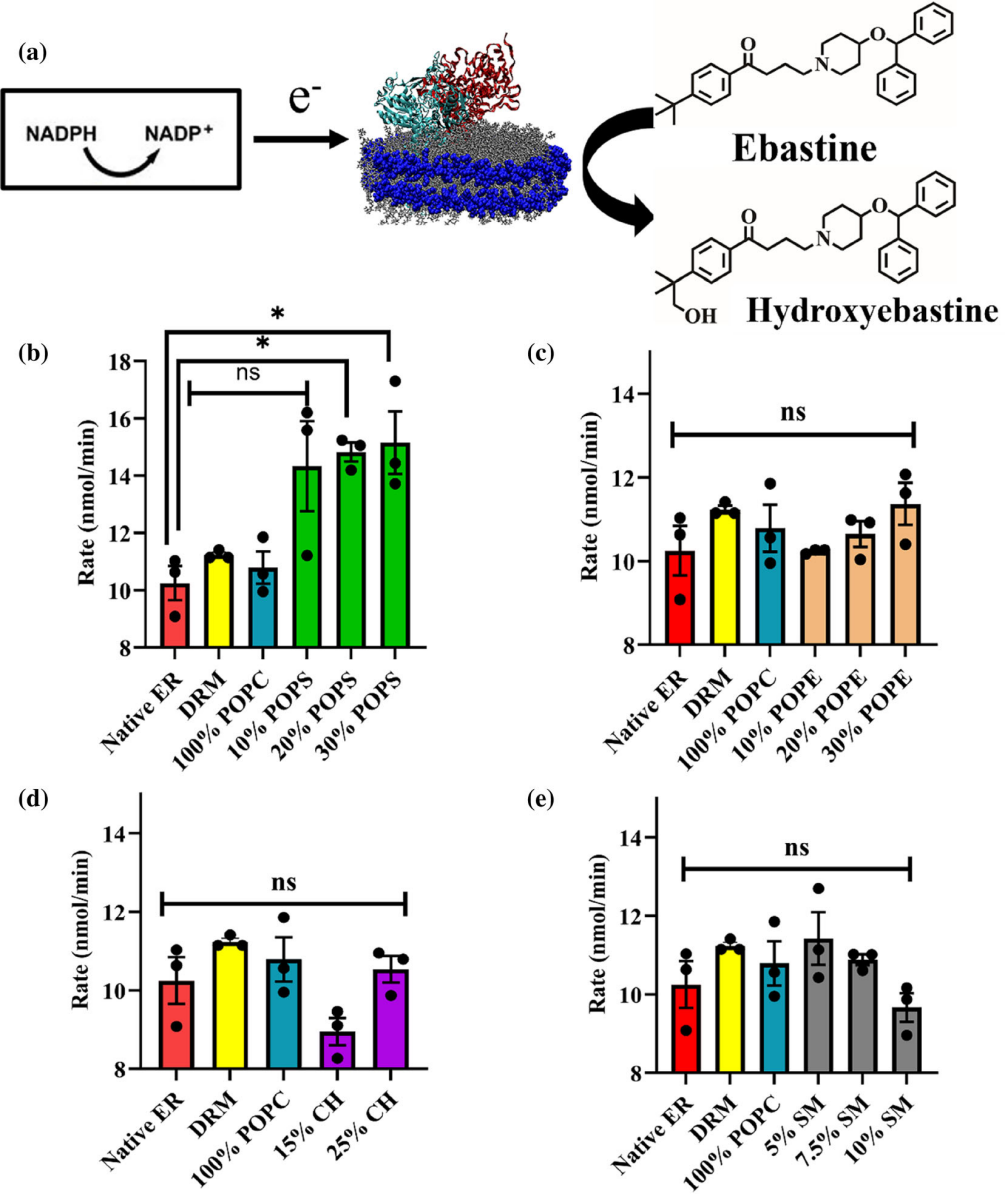
(a) Schematic of NADPH oxidation and ebastine hydroxylation by a CPR‐CYP2J2 Nanodisc complex. CPR (cyan) is added to solution separately and self‐associates with the lipid bilayer. Electrons are shuttled from NADPH to CYP2J2 (in red) via cytochrome P450 reductase redox centers. These electrons subsequently drive CYP2J2 P450 catalysis of ebastine to hydroxyebastine. Rate of NADPH oxidation (nmol/min) for (b) POPC‐POPS (c) POPC‐POPE (d) POPC‐cholesterol and (e) POPC‐sphingomyelin nanodiscs. Statistical analysis performed using Brown‐Forsythe and Welch One‐way ANOVA test. All data is compared to native ER. * = *p* <0.05. SEM is calculated from measurements in triplicates.

Cytochrome P450 Reductase (CPR), the redox partner to P450 enzymes, is responsible for transferring electrons from NADPH to P450s, allowing them to oxidize their substrates and convert NADPH to NADP+ in the process. This electron transfer is important in the catalytic cycle of P450s. Since NADPH gets converted to NADP+ during the reaction, the decrease in absorbance of NADPH at 340 nm over a period of 10 min was measured to calculate the rate of consumption of NADPH, which is equivalent to the rate of electron transfer from CPR to CYP. Figure 6 shows the variation in rates (in nmol/min) of NADPH utilization for all the CYP2J2 NDs.

As is evident, the electron transfer rate between CYP and CPR is impacted due to changes in lipid composition. Interestingly, increasing amounts of the negatively charged POPS led to a steady increase in the rate of electron transfer between CYP‐CPR. Among all 14‐lipid composition NDs, we see the highest rates for 30% POPS NDs. This underscores the importance of the charge of the lipid headgroup in mediating the enzyme's functional properties.

For POPE and CH containing NDs, we also observe an increase in electron transfer rates with increasing lipid concentration in the nanodiscs. However, for SM NDs, as SM% increases, electron transfer rates decrease steadily. On comparison across the 14‐lipid mixed nanodiscs, we see that 30% POPS NDs show the maximum and 15% CH, and 10% SM NDs show the minimum rates of oxidation of NADPH (Figure 6).

Analyzing the trends observed in thermostability and NADPH oxidation rates, we see that as POPS% increases, the enzyme becomes less thermostable, while the CYP‐CPR electron transfer rates increase. This effect is also observed for POPE and CH NDs. However, for SM NDs, increasing SM content corresponds to greater thermostability and reduced electron‐transfer rates. Overall, from nanoDSF measurements and the NADPH oxidation assay, we see opposite trends for thermostability and CYP‐CPR electron transfer.

### 2.6 Lipid composition does not affect the substrate binding to CYP2J2 in nanodisc

**FIGURE 7:**
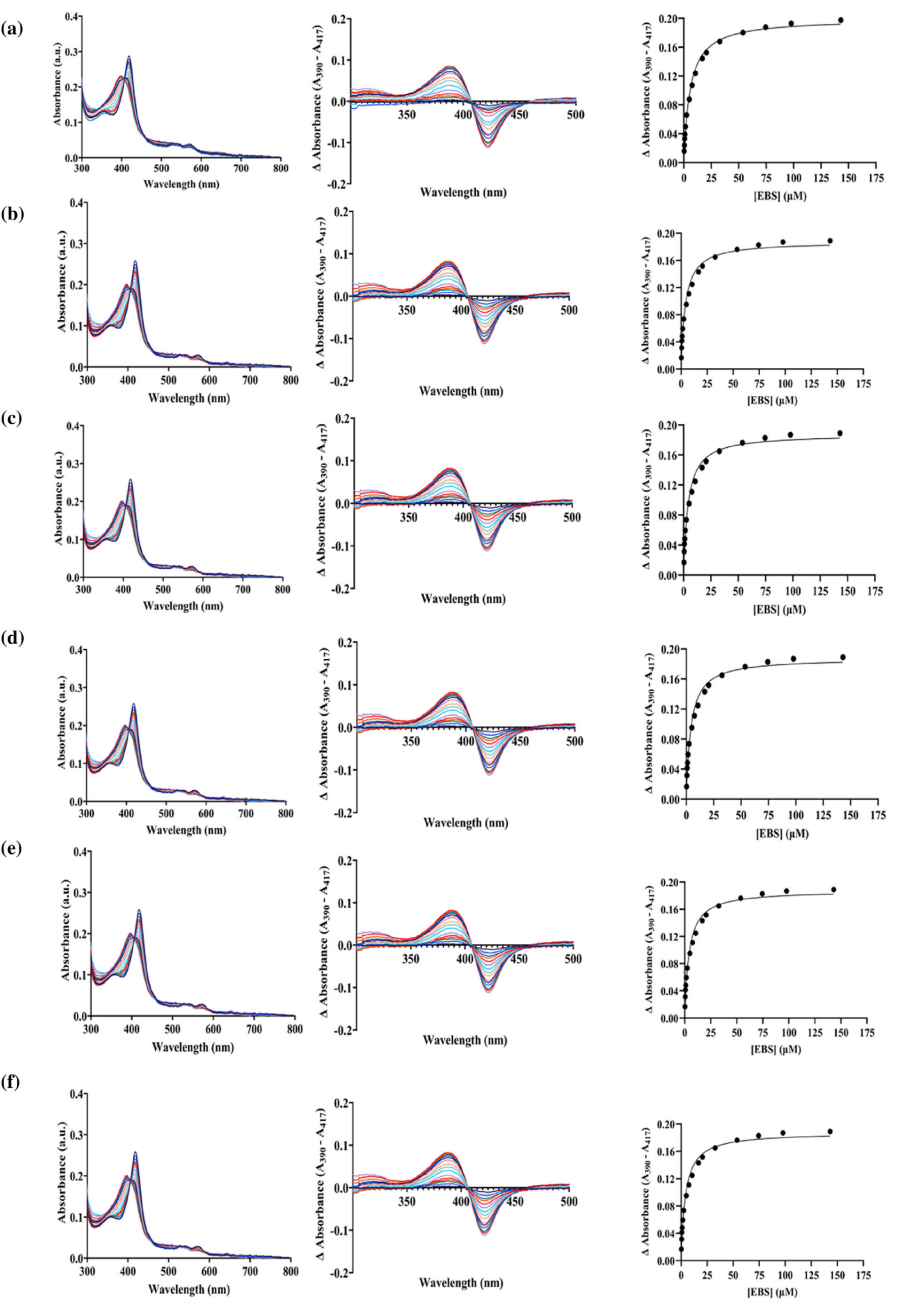
Effect of lipid composition on binding of CYP2J2 in nanodiscs with ebastine. Raw spectra, difference spectra, and delta absorbance (A_390_–A_417_) plotted against ebastine concentration for (a) 70% POPC + 30% POPS, (b) 90% POPC + 10% sphingomyelin, (c) 75% POPC + 25% CH, (d) 70% POPC + 30% POPE, (e) Native ER nanodiscs, and (f) CYP2J2 in detergent micelles (cholate). Ebastine was added incrementally from 0 to 143 μM. Ebastine‐binding spectra were fitted to a single binding isotherm, and K_d_ was calculated for all conditions.

To study whether lipid composition affected substrate binding, binding studies of CYP2J2 in nanodiscs of different lipid compositions were carried out with Ebastine (0.25–143 μM). Ebastine, an antihistaminic drug, is a known substrate of CYP2J2, which shows a Type 1 shift upon binding (McDougle et al., 2013). The interaction of Ebastine with the enzyme displaces water molecules from the active site and induces a transition in the iron spin state from a lower‐spin state to a higher‐spin state. This spin‐state shift results in a blue shift of the maximum absorption wavelength from 417 nm to ~390 nm. The change in absorbance is plotted against increasing substrate concentration and fitted into a substrate‐binding equation to calculate the binding affinity. We conducted binding studies for the following: 70% POPC + 30% POPS, 90% POPC + 10% sphingomyelin, 75% POPC + 25% CH, 70% POPC + 30% POPE, Native ER and CYP2J2 in detergent micelles (cholate). The respective K_d_ values for the different lipid composition NDs are listed in Table 3. As shown in Figure 7 and Table 3, ligand binding affinities do not change drastically across the lipid compositions. These results demonstrate that lipid composition does not affect the functional properties of CYP2J2 nanodisc by affecting substrate binding to the enzyme.

**TABLE 3:** Dissociation constants of binding of CYP2J2 in nanodiscs of different lipid compositions with Ebastine.

Nanodisc	K_D_ (μM)
30% POPS	5.66 ± 0.76
30% POPE	5.02 ± 0.43
25% CH	4.01 + 0.50
10% SM	4.11 ± 0.29
Native ER	6.78 ± 0.30
CYP2J2 (micelles)	7.2 ± 0.53

### 2.7 Lipid composition changes the fluidity of the membrane

**FIGURE 8:**
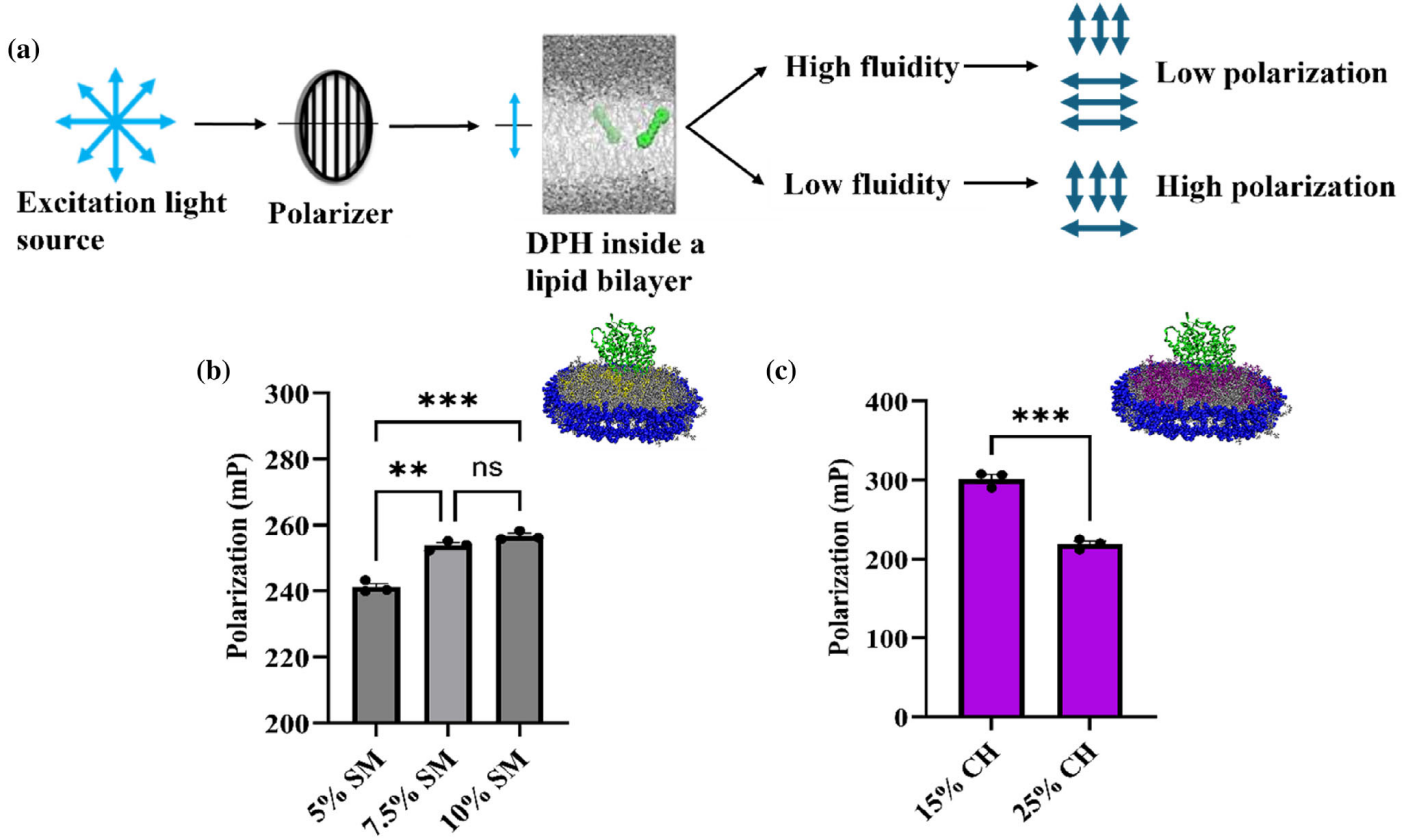
Characterizing the mixed lipid nanodiscs based on their membrane fluidity. (a) Schematic for fluorescence polarization measurements involving 1,6‐diphenyl‐1,3,5‐hexatriene (DPH). DPH inserts itself into the hydrophobic regions of the lipid bilayer. In more fluidic membranes, the dye has more rotational freedom leading to more depolarized fluorescence emission. Fluorescence polarization measurements for (b) POPC‐sphingomyelin and (c) POPC‐cholesterol nanodiscs. For sphingomyelin nanodiscs, with increasing sphingomyelin content, polarization increases and membrane fluidity decreases. Whereas, with increasing cholesterol content, polarization decreases and membrane fluidity increases. This underlines the possible correlation between membrane fluidity and function of CYP2J2 in nanodiscs. Statistical analysis performed using Brown‐Forsythe and Welch One‐way ANOVA test for (b) and paired t test for (c). * = *p* <0.05; ** = *p* <0.01; *** = *p* <0.001. All measurements have been done in triplicates and SEM has been plotted as error bars.

While NDs containing varying degrees of POPS lipid showing high activity and electron transfer rates can be attributed to the negative charge of the lipid head‐group that helps in stabilizing the CYP‐CPR complex via charge interaction, the same rationale does not apply to cholesterol and sphingomyelin containing NDs, since these lipid headgroups have a net neutral charge. However, both lipids have been implicated in modulating the membrane's overall fluidity. In addition to the lipids, nanodiscs have been shown to alter membrane fluidity. Molecular and scattering studies of nanodiscs revealed that the ordering of lipids within the disk is significantly heterogeneous and distinct from that of large vesicles. In particular, lipid ordering varies between the nanodisc center and rim, and interactions with the scaffold protein can alter the gel/liquid phase behavior of the lipids relative to vesicles, indicating that nanodisc constraints affect bilayer physical properties, including fluidity (Bengtsen et al., 2020) (Her et al., 2016). Additionally, it has been demonstrated that confinement by the MSP in nanodiscs alters lipid packing, increases acyl‐chain order, and suppresses collective bilayer fluctuations, resulting in reduced membrane fluidity and physicochemical properties that differ fundamentally from those of extended bilayers (Denisov et al., 2005) (Dickey & Faller, 2008). To understand how the presence of lipids like CH and SM in the composition, coupled with the use of nanodiscs as a mimic, influences bilayer fluidity, we characterized the mixed lipid nanodiscs containing CYP2J2 through Fluorescence Polarization (FP) measurements using 1,6‐Diphenyl‐1,3,5‐hexatriene (DPH) (Figure 8a). DPH is a hydrophobic fluorescent dye that partitions itself in the inner hydrophobic regions of the lipid bilayer (Poojari et al., 2019). We used DPH to report on the effect of lipid composition on membrane fluidity. In more fluid bilayers, these dyes have larger rotational freedom, leading to a loss in polarization. Therefore, polarization is inversely correlated to membrane fluidity. The CYP2J2 NDs were stained with DPH by incubating at room temperature for 1 h in the dark with mild rocking. The polarization measurements were taken by exciting the fluorophore at 360 nm and recording the emission at 430 nm. For SM NDs, we observed an increase in the polarization values with increasing SM content in the bilayer (Figure 8b). This meant that increasing SM percentages decreased overall membrane fluidity. This can be explained by the fact that SM has saturated fatty acyl chains, which are known to pack more tightly. As shown in Figure 8c, for CH NDs, DPH showed that, as the CH percentage increased, the membrane's overall fluidity also increased. Taken together, these results show that the addition of SM and CH has opposing effects on the membrane fluidity. These results with DPH also suggest a possible direct correlation between membrane fluidity and the rate of electron transfer between CYP and CPR.

## 3 DISCUSSION

The unfolding of P450 enzymes has been studied previously using chemical denaturants, thermal stress, hydrostatic pressure, organic solvents, and other methods (González‐Pérez et al., 2012; Maves & Sligar, 2001; Yu et al., 1995). Previously, DSC measurements have shown that a collection of human microsomal P450s exhibits two major transitions during unfolding. The first transition is shown to be broad, with several composite peaks, including one around 50°C and another around 60**°**C (Anzenbacher et al., 1982). This is close to our observation that the first two transitions of CYP2J2 in detergent micelles occur at around 50 and 60**°**C. We observe the third transition for CYP2J2 in detergent micelles at around 75°C, rather than at around 90**°**C (Anzenbacher et al., 1982). This can be explained by the fact that the previous study did not use a specific isoform but instead used a collection of different P450 isoforms. Unfolding studies have also been done on CYP3A4‐nanodiscs. CYP3A4 NDs showed a major transition peak at around 50**°**C with three smaller transitions observed, with T_m_ values of 46, 53, and 56°C. The presence of smaller transitions within the major transition peak is consistent with previous studies and further validates the appearance of two peaks around 50–60°C for CYP2J2. In a separate study using nanoDSF, CYP3A4 in NDs was found to have a T_m_ of ~59°C (Knetsch & Ubbink, 2024). While neither of these studies reported a second transition for CYP3A4, we observe a second dominant transition for CYP2J2 in NDs at around 75**°**C. Similarly, DSC thermograms have shown CYP119 unfolding through two major transitions (Maves & Sligar, 2001). This underscores that unfolding behavior and the transition states are CYP isoform dependent. For all the nano‐DSF first‐derivative plots of CYP2J2 in detergent and in nanodiscs, we observe a transition around 75**°**C with a more prominent peak than the transition around 50**°**C. Since the derivative plot (d(F350/F330)/dT) shows the rate of change of the fluorescence ratio with temperature, a taller peak would correspond to more buried tryptophan residues becoming solvent‐exposed. This suggests that most of the protein unfolds at 75°C, while only a minority of the population unfolds around 50°C.

For a bacterial disulfide‐bridge‐forming protein, the shift from micelle to nanodisc environment has been shown to alter the protein's unfolding behavior (Selvasingh et al., 2024). For CYP2J2, we also observe a shift in the unfolding pattern between detergent micelles and the nanodisc environment, as evidenced by the first‐derivative plots in nano‐DSF. When CYP2J2 is incorporated into a nanodisc, the second transition around 60**°**C becomes less prominent and no longer remains an individual peak. Previously, the presence of multiple melting transitions is attributed to different domains within the enzyme (Anzenbacher et al., 1982). In DSC thermograms and nano‐DSF first derivative plots, the appearance of a sharp peak is usually indicative of a highly cooperative transition. The individual peak around 60**°**C for detergent micelles appears minor and merges with the larger peak at around 75**°**C for CYP2J2 in NDs. It has been suggested that membrane incorporation can suppress intermediate unfolding transitions by thermodynamically coupling domains, leading to a more globally cooperative unfolding. The unfolding pathway, therefore, becomes more two‐state‐like (fewer distinct cooperative domains) (Ferguson‐Miller & Sligar, 2018; Fitter, 2003; Martinis et al., 1996; Maves & Sligar, 2001; Eftink, 1994; Murugan & Mazumdar, 2006; Privalov, 1979). This supports the observation that the unfolding of the enzyme is sensitive to its immediate membrane environment that alters the unfolding pattern of membrane proteins.

We found that as POPS content (negative charge of the lipid headgroup) in the membrane lipid composition increases, the rate of electron transfer increases, with 30% POPS NDs showing the maximum rate among the 14 lipid compositions tested. Previously, replacement of PC with PS was found to stimulate 7‐ethoxycoumarin and *p*‐nitroanisole metabolism for CYPLM2 (Ingelman‐Sundberg et al., 1981). Additionally, the rate of ebastine hydroxylation by CYP2J2 increased with the increase in the percentage of POPS in the nanodisc lipid membrane (Huff et al., 2019; Meling et al., 2015). Previously, it has been shown that the presence of anionic phospholipids enhances protein insertion into the lipid bilayer for CYP1A2, CYP3A4, CYP2B1, and CYP2J2 (Ahn et al., 1998) (Kim et al., 2003) (Kim et al., 2007) (McDougle et al., 2015). Through MD simulations and electrostatic mapping of the charged residues in CYP2J2, we identified the residues responsible for attachment to the lipid bilayer to lie predominantly in a positively charged region of the protein's structure (Figure S3). This validates the observation that the presence of anionic phospholipids aids protein insertion into the membrane by aiding favorable electrostatic interactions between the protein and the lipids in the bilayer. Previously, it was shown that anionic lipids enhance the activity of the Cytochrome b6f complex by binding to a designated phospholipid binding site and facilitating the electron transfer (Bhaduri et al., 2019). Lastly, it has been shown that with the addition of 50% PS to PC containing NDs, the redox potential of CPR became more negative. Therefore, CPR has a higher tendency to donate electrons to the CYP and initiate its catalytic cycle in the presence of anionic lipids (Das & Sligar, 2009). Therefore, the presence of negatively charged phospholipids results in a higher rate of electron transfer, greater interaction between CYP‐CPR, and enhanced catalytic activity.

We observe that the trends in thermal stability and NADPH oxidation rates across all 14 lipid compositions are opposite (Table 2 and Figure 6). While POPS NDs have the highest electron‐transfer rates due to the negative charge of the lipid headgroup, increasing POPS content might lead to destabilizing electrostatic repulsive interactions arising from the high charge density in the ND assembly. For SM NDs, the same phenomenon can be rationalized through the concept of lipid packing order and dynamics within the membrane. In reconstituted HDL complexes, fully saturated phosphatidylcholines (PCs) give greater thermal stability (Guha et al., 2008). Similarly, saturated lipid bilayers with added cholesterol show higher rupture tension and more mechanical strength (Lyu et al., 2020). Recently, CYP3A4 was shown to be thermostabilized by incorporation into DPhPC (a phospholipid with a branched fatty acyl chain, leading to tighter packing) containing NDs (Knetsch & Ubbink, 2024). Overall, it becomes clear that lower membrane fluidity and tighter packing generally lead to greater resistance to thermal stress.

Reduced membrane fluidity and tighter lipid packing can also negatively affect the functional dynamics and electron‐transfer efficiency between P450 enzymes and CPR in nanodiscs. Productive CYP–CPR electron transfer depends on transient but specific interactions that are mediated by the lateral mobility and dynamic conformational alignment of both proteins within the membrane. When the bilayer becomes more ordered, such as in the presence of saturated phospholipids or SM, the reduced fluidity limits lateral diffusion and the probability of CYP‐CPR encounters (Bridges et al., 2020; Šrejber et al., 2018). Moreover, tighter lipid packing constrains the conformational freedom of the F–G loop and N‐terminal anchor of CYPs, as well as the FMN‐binding domain of CPR, both of which require flexibility to achieve the correct orientation for electron tunneling (Bayburt et al., 2011; Cojocaru et al., 2011). Computational and biophysical studies have shown that the membrane composition can modulate the redox potential of CPR and influence CYP heme orientation and insertion depth, thereby altering the heme–FMN distance critical for efficient electron transfer (Campbell et al., 2014; Otyepka et al., 2012). Consequently, in nanodiscs with rigid or tightly packed lipids, decreased membrane dynamics lead to suboptimal CYP–CPR complex formation and reduced catalytic turnover, reflecting the essential role of a moderately fluid membrane environment in maintaining efficient P450 redox cycling.

In conclusion, lipid composition was found to modulate the thermal and functional properties of CYP2J2 in nanodiscs, potentially through electrostatic interactions involving the charge of the lipid headgroup, as well as altering membrane fluidity.

## 4 METHODS

### 4.1 Expression and purification of CYP2J2

The CYP2J2 construct with N‐terminus truncation and a penta‐His tag added at the C‐terminus (M2D34G construct) was used for this study (McDougle et al., 2013). CYP2J2 was grown and purified using the following protocol:

Double transformant colonies containing the CYP2J2 gene and pTGro7 gene were cultured in 30 mL of Luria Bertani (LB) media with chloramphenicol (20 μg/mL) and ampicillin (100 μg/mL) at 37°C and 220 rpm overnight. 5 mL of this culture was used to inoculate 500 mL of Terrific Broth (TB) media supplemented with trace elements, ampicillin (100 μg/mL), and chloramphenicol (20 μg/mL). The culture was grown for 2.5 h at 37°C and 220 rpm. Delta‐aminolevulinic acid (500 mL of 0.5 mM) was then added, and the culture was grown for another 2 h at 26°C and 160 rpm. After reaching a target absorption (A_600_ = 1.2), 1 mM of IPTG was added to induce protein expression. In addition, 2 g of arabinose was added to each 500 mL culture. The cultures were grown for 44 h, and then the cells were harvested by centrifugation at 9550 RCF for 15 min at 4°C. The cell pellet was resuspended in buffer 1 (100 mM potassium phosphate pH 7.4, 20% glycerol, 6 mM magnesium chloride, 5 mM beta‐mercaptoethanol (βME), and 0.2 mM phenylmethanesulfonylfluoride (PMSF), 1 mg of DNase). The cells were sonicated on ice for 6 cycles of 40 s with breaks of 40 s at an amplitude of 80%. This was followed by centrifugation at 95834 RCF at 4°C for 45 min. The membrane fraction pellet was resuspended in a solubilization buffer 2 (100 mM potassium phosphate pH 7.4, 20% glycerol, 200 mM sodium chloride, 5 mM beta‐mercaptoethanol (βME), and 1% cholate) for 3–5 h at 4°C. This was further centrifuged at 95834 RCF at 4°C for 45 min to obtain the supernatant, which contained the target membrane protein. The supernatant was then loaded onto a Ni‐NTA column equilibrated with the column buffer 3 (100 mM potassium phosphate pH 7.4, 20% glycerol, and 0.1% cholate and 0.5 mM βME). It was further washed with four column volumes of ATP‐containing buffer 4 (100 mM potassium phosphate pH 7.4, 20% glycerol, 0.1% cholate, 5 mM ATP, 10 mM magnesium chloride, and 150 mM potassium chloride) to remove the GroEL that may be bound to the CYP protein. The column was washed with five column volumes of the wash buffer (100 mM potassium phosphate pH 7.4, 20% glycerol, 0.1% cholate, and 20 mM imidazole). Finally, the protein was eluted with four column volumes of the elution buffer (100 mM potassium phosphate pH 7.4, 20% glycerol, 0.1% cholate, and 200 mM imidazole). The eluted protein was concentrated and buffer exchanged to remove imidazole using Amicon centrifugal filters (30,000 MWCO) and stored at −80°C.

### 4.2 Expression and purification of dark MSP 1D1

The previously modified ΔTrp‐MSP1D1 (Dark MSP 1D1) construct was used for this study (McLean et al., 2020) (McDougle et al., 2015). MSP was expressed and purified as described previously (Denisov et al., 2004).

### 4.3 Reconstitution of CYP2J2 in nanodiscs of different lipid composition

Purified CYP2J2 was reconstituted in nanodiscs of different lipid compositions according to Table 1. The lipids were dissolved in chloroform to make stocks and quantified using the Phosphate assay (wherever appropriate). Following this, the lipids were taken in appropriate amounts, dried down using nitrogen gas, and then in a vacuum desiccator overnight. After resolubilizing the lipids using 200 mM cholate, dark MSP1D1 was added (MSP:lipid ratio of 1:140), and the mixture was allowed to mix at 4°C with mild rocking for 1 h. CYP2J2 was then added (1:15 CYP2J2:MSP ratio) to the mixture and incubated again at 4°C with mild rocking for 1 h. To aid the spontaneous assembly of the CYP2J2‐NDs by removal of the detergent, Amberlite XAD beads were added to the final mixture and incubated with mild rocking for 7 h at 4°C. The mixture was separated from the beads by filtration, and the resulting solution was concentrated, loaded onto a Superdex 200 10/300 column for Size Exclusion Chromatography, and purified using the Cytiva ÄKTA™ go protein purification system. Absorbance at 280 nm was used to identify the peak corresponding to CYP2J2‐NDs, and the collected fractions were concentrated and quantified at 417 nm using a Shimadzu UV‐2700i UV–Visible spectrophotometer. The successful incorporation of CYP2J2 in nanodiscs was verified by SDS‐PAGE showing two distinct bands for CYP2J2 and dark MSP1D1. Glycerol was added to a final concentration of 10% v/v to the aliquots and stored at −80°C for further studies.

### 4.4 Thermal stability analysis of CYP2J2 in nanodiscs using nano‐DSF

Nano‐DSF measurements were carried out using a Prometheus NT.48 nano‐DSF instrument (NanoTemper Technologies). The samples were loaded using glass capillaries and placed adjacent to each other in the sample loading tray. The samples were subjected to a temperature range of 20–90**°**C with a thermal ramp of 1**°**C/min and all measurements were done in triplicates. Samples were excited at 280 nm and the intrinsic tryptophan fluorescence at 350 nm and 330 nm were recorded as a function of temperature to monitor changes upon thermal unfolding. For data analysis, the F350/F330 ratios and the experimental first derivatives for each triplicate were exported from the Prometheus Panta Control software.

### 4.5 Unfolding studies of CYP2J2 using UV‐spectrophotometry

200 μL of CYP2J2 ND made with the reported native ER lipid composition (as discussed in Table 1) was subjected to the following temperature points: 30, 35, 40, 42.5, 45, 47.5, and 50**°**C and was allowed to equilibrate to the temperature for 5 min. The ratio of absorbance at 360 nm to absorbance at 417 nm was plotted as a function of temperature to report on the fraction of heme dissociation. In a separate experiment, CYP2J2 ND was subjected to a temperature of 50**°**C, and the absorbance at 360 nm was measured separately across a period of 10 min. The data was recorded on a Shimadzu UV‐2700i UV‐Visible spectrophotometer.

### 4.6 Thermal unfolding studies of CYP2J2 in nanodiscs using CO‐binding assay

200 μL of CYP2J2 ND made with the reported native ER lipid composition was incubated at four temperatures: 25, 50, 60 and 75**°**C for 10 min each. The CO‐binding assays were performed according to the previously published protocol (Arendse & Blackburn, 2018) (Meling et al., 2015).

Readings from the CO‐binding spectra were used to calculate the percentage conversion of P450 to P420 according to previous literature (Arendse & Blackburn, 2018).

### 4.7 Binding of CYP2J2 in nanodiscs with Ebastine using UV‐spectrophotometry

The protocol for spectroscopic binding analysis was adapted from previous literature (McDougle et al., 2013). The data were recorded on a Shimadzu UV‐2700i UV‐Visible spectrophotometer. The recorded data were analyzed on GraphPad Prism 10 and fitted to a substrate binding isotherm equation as follows:

$$Y=\frac{B_{\max}*\left[L\right]}{K_{d}+\left[L\right]}$$

[*L*] is the concentration of the ligand. B_max_ is the maximum number of binding sites, expressed in the same units as the Y‐axis. *K*_d_ is the equilibrium dissociation constant, expressed in the same units as the X‐axis (concentration).

### 4.8 NADPH oxidation assay

All reactions were carried out by equilibrating 0.2 μM CYP2J2‐ND, 0.6 μM CPR, and 40 μM ebastine (solubilized in DMSO) in a buffered solution of 100 mM potassium phosphate (pH = 7.4) at 37**°**C for 3 min. Following the incubation, the reaction was initiated at t = 0 min by the addition of 200 μM NADPH (solubilized in ddH2O) to reach a final reaction volume of 400 μL. NADPH absorption was measured at 340 nm for a period of 10 min using a Shimadzu UV‐2700i UV‐Visible spectrophotometer in kinetics mode (Huff et al., 2019).

### 4.9 Steady‐state FP measurements with DPH

DPH was dissolved in DMSO to prepare a stock solution. CYP2J2 NDs (3 μM) were stained with DPH in a final ratio of 1:200 (DPH: lipid) mol ratio for 1 h at room temperature in the dark with mild rocking. Steady‐state fluorescence polarization measurements (FP) were taken by exciting DPH at 360 nm and recording the emission at 430 nm (BMG Labtech, Clariostar Plus). The FP was calculated according to the following equation:

$$\text{Fluorescence Polarization}\;\left(\mathrm{FP}\right)=I_{\mathrm{II}}-Iꓕ/I_{\mathrm{II}}+Iꓕ;$$

where I_II_ and Iꓕ are fluorescent intensities measured in the parallel and perpendicular channels, respectively (De Santis et al., 2018; Poojari et al., 2019).

## AUTHOR CONTRIBUTIONS

**Rajatabha Das:** Investigation; writing – original draft; methodology; validation; writing – review & editing; formal analysis; conceptualization; supervision; data curation; software. **Henry M. Mastrion:** Methodology; data curation; formal analysis; software; writing – review & editing. **Harrison B. Vassar:** Methodology; data curation; writing – review and editing; formal analysis; software. **Aditi Das:** Conceptualization; Investigation; funding acquisition; writing – original draft; methodology; validation; writing – review and editing; formal analysis; supervision.

## CONFLICT OF INTEREST STATEMENT

The authors declare no conflicts of interest.

## Supporting information


**Figure S1.** Effect of lipid composition on thermal stability of CYP2J2 in nanodisc. Ratio of fluorescence intensity at 350 nm and 330 nm (F_350_/F_330_) plotted against a thermal ramp from 20**°**C to 90**°**C for CYP2J2 in (A) POPC‐POPE (B) POPC‐cholesterol (C) POPC‐sphingomyelin (D) Native ER and DRM nanodiscs. Average of triplicates has been plotted for all plots.
**Figure S2.** Purification and characterization of dark MSP1D1. (A) UV‐Visible spectrum (250–800 nm) of dark MSP1D1 showing a peak at 280 nm. (B) Snapshot from the discovery scan function in the Prometheus NanoDSF instrument showing a comparison between the raw fluorescence of a fluorescent MSP construct and dark MSP1D1 construct. As evident, the dark MSP1D1 shows negligible fluorescence contribution. This justifies the use of this construct for all our nanoDSF measurements.
**Figure S3.** Surface projection of electrostatic mapping of CYP 2 J2 showing the regions with positive electrostatic potential (Blue) and negative electrostatic potential (Red). Residues involved in binding with the lipid bilayer are located in a positively charged region of the protein. This points to possible electrostatic interactions between the protein and the negatively charged lipids in the membrane leading to more insertion depth of CYP2J2 in the lipid bilayer with POPS lipids.
**Figure S4.** (A) Fluorescence intensity of CYP2J2 ND (100% POPC) at 330 and 350 nm with increasing temperature showing a gradual decrease in the intensity due to thermal quenching and (B) Emission spectrum of CYP2J2 ND (100% POPC) at 30**°**C and 55**°**C showing the shift in emission peak wavelength from 330 nm to 350 nm.
**Figure S5.** (A) Raw spectra (300 nm‐800 nm) for CYP2J2 ND made with native ER composition incubated at discrete temperatures (30–55**°**C) for 10 min; (B) Ratio of absorbances at 360 nm and 417 nm (A_360_/A_417_) plotted against temperature show increasing heme perturbation as a consequence of thermal denaturation. Average of triplicates is plotted with SEM as error bars.

## Data Availability

The data that support the findings of this study are available from the corresponding author upon reasonable request.
