# Supplementary material for: Differential effects of lipid composition on the thermal and functional properties of membrane associated CYP2J2
Source: Protein Sci. 2026 Jun 6;35(7):e70654. doi: 10.1002/pro.70654 (PMC13242164; doi:10.1002/pro.70654)
Supplement: Supplementary file 1 — Figure S1. Effect of lipid composition on thermal stability of CYP2J2 in nanodisc. Ratio of fluorescence intensity at 350 nm and 330 nm (F350/F330) plotted against a thermal ramp from 20°C to 90°C for CYP2J2 in (A) POPC‐POPE (B) POPC‐cholesterol (C) POPC‐sphingomyelin (D) Native ER and DRM nanodiscs. Average of triplicates has been plotted for all plots. Figure S2. Purification and characterization of dark MSP1D1. (A) UV‐Visible spectrum (250–800 nm) of dark MSP1D1 showing a peak at 280 nm. (B) Snapshot from the discovery scan function in the Prometheus NanoDSF instrument showing a comparison between the raw fluorescence of a fluorescent MSP construct and dark MSP1D1 construct. As evident, the dark MSP1D1 shows negligible fluorescence contribution. This justifies the use of this construct for all our nanoDSF measurements. Figure S3. Surface projection of electrostatic mapping of CYP 2 J2 showing the regions with positive electrostatic potential (Blue) and negative electrostatic potential (Red). Residues involved in binding with the lipid bilayer are located in a positively charged region of the protein. This points to possible electrostatic interactions between the protein and the negatively charged lipids in the membrane leading to more insertion depth of CYP2J2 in the lipid bilayer with POPS lipids. Figure S4. (A) Fluorescence intensity of CYP2J2 ND (100% POPC) at 330 and 350 nm with increasing temperature showing a gradual decrease in the intensity due to thermal quenching and (B) Emission spectrum of CYP2J2 ND (100% POPC) at 30°C and 55°C showing the shift in emission peak wavelength from 330 nm to 350 nm. Figure S5. (A) Raw spectra (300 nm‐800 nm) for CYP2J2 ND made with native ER composition incubated at discrete temperatures (30–55°C) for 10 min; (B) Ratio of absorbances at 360 nm and 417 nm (A360/A417) plotted against temperature show increasing heme perturbation as a consequence of thermal denaturation. Average of triplicates is plotted with SEM as error ba [file PRO-35-e70654-s001.docx]

**Differential Effects of Lipid Composition on the Thermal and Functional Properties of Membrane Associated CYP2J2**

*Rajatabha Das^1^, Henry M Mastrion^2^, Harrison B Vassar^2^ and Aditi Das^1*^*

*^1^School of Chemistry and Biochemistry, College of Sciences, Parker H. Petit Institute for Bioengineering and Biosciences*

** Corresponding Author. Prof. Aditi Das, aditi.das@chemistry.gatech.edu*

Table of Contents

[1. NanoDSF ratio plots for all mixed-lipid CYP2J2 nanodiscs 3](#_Toc223208136)

[2. Fluorescence contribution from dark MSP 1D1 4](#_Toc223208137)

[3. Electrostatic mapping of CYP2J2 structure 5](#_Toc223208138)

[4. Control experiments for Nano-DSF 6](#_Toc223208139)

[5. Thermal unfolding of CYP2J2 ND using UV-Visible Spectroscopy 7](#_Toc223208140)

# **NanoDSF ratio plots for all mixed-lipid CYP2J2 nanodiscs**


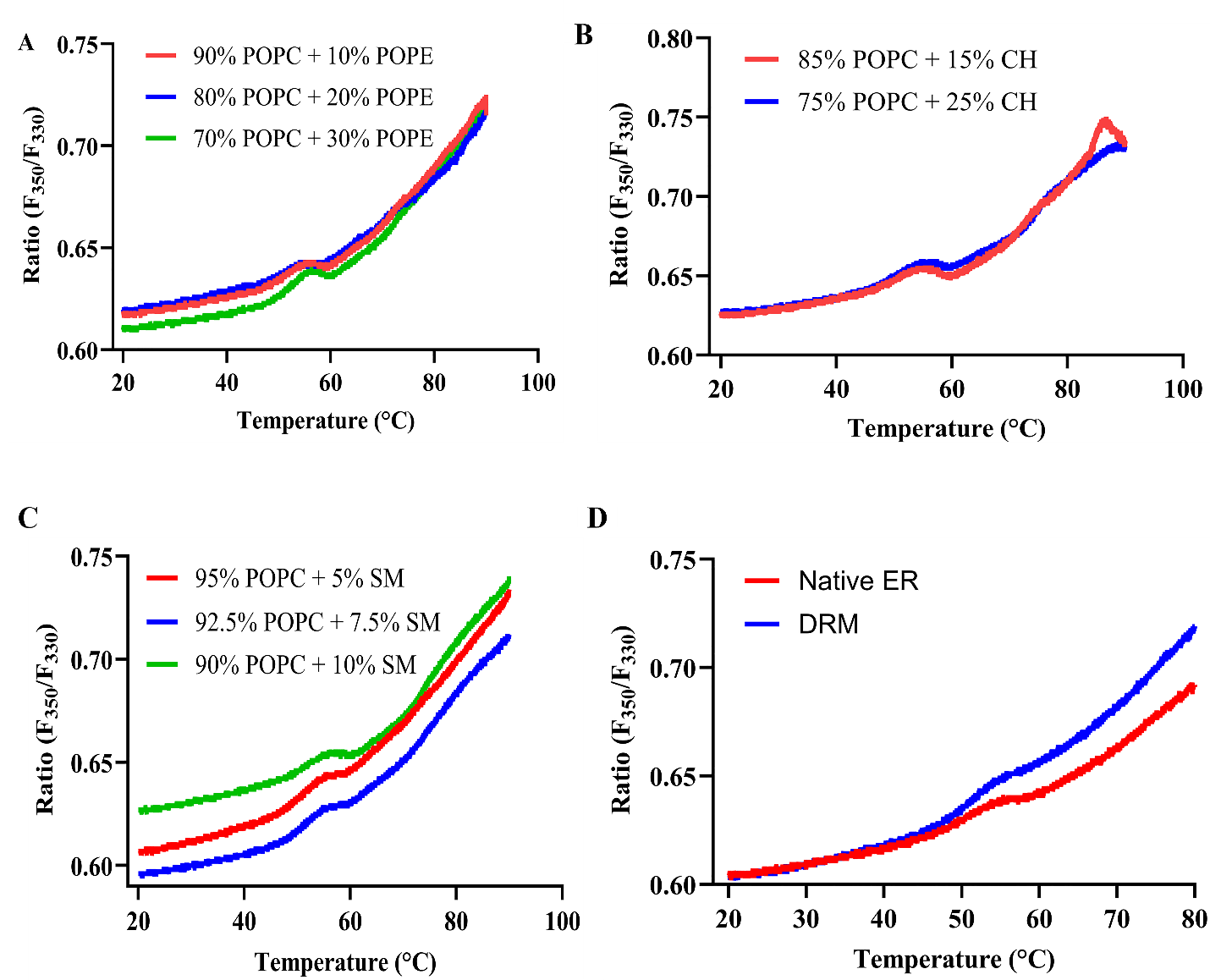


**Figure S1.** Effect of lipid composition on thermal stability of CYP2J2 in nanodisc. Ratio of fluorescence intensity at 350 nm and 330 nm (F_350_/F_330_) plotted against a thermal ramp from 20**°**C to 90**°**C for CYP2J2 in (A) POPC-POPE (B) POPC-cholesterol (C) POPC-sphingomyelin (D) Native ER and DRM nanodiscs. Average of triplicates has been plotted for all plots.

# **Fluorescence contribution from dark MSP 1D1**


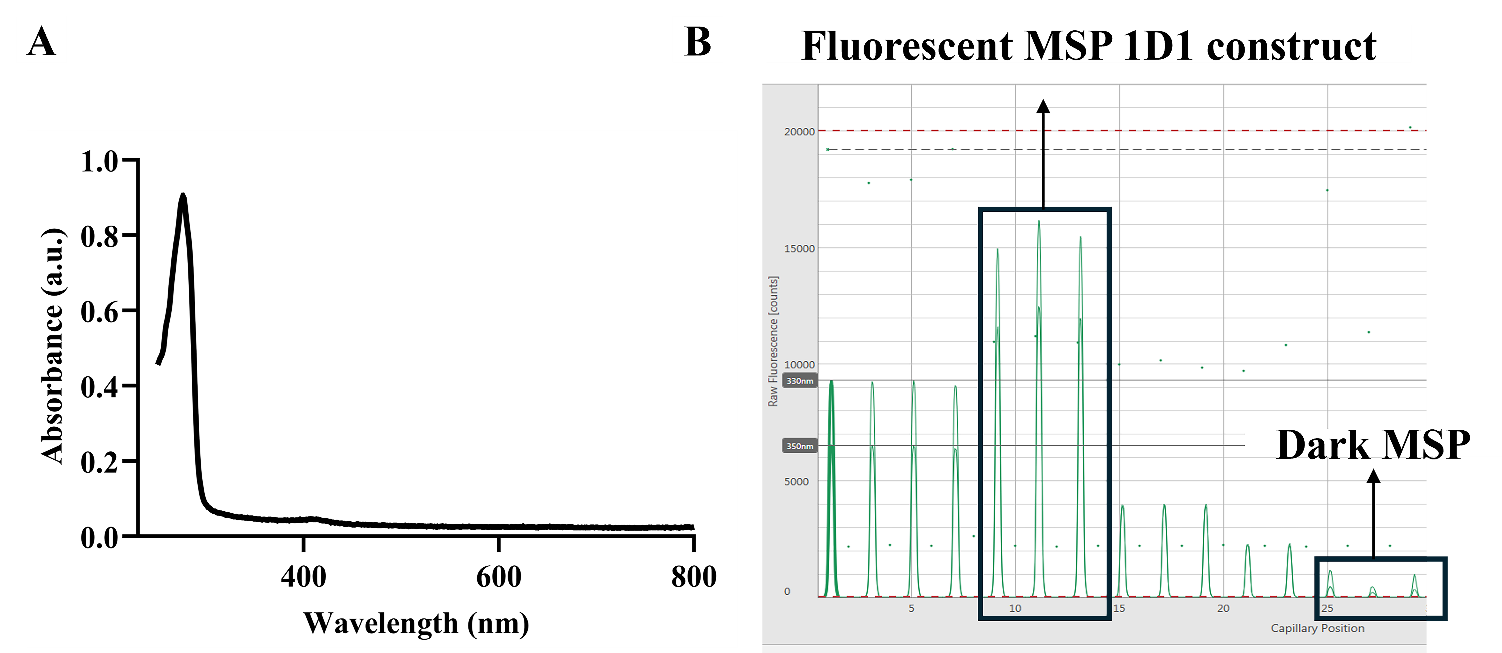


**Figure S2.** Purification and characterization of dark MSP1D1. (A) UV-Visible spectrum (250 nm–800 nm) of dark MSP1D1 showing a peak at 280 nm. (B) Snapshot from the discovery scan function in the Prometheus NanoDSF instrument showing a comparison between the raw fluorescence of a fluorescent MSP construct and dark MSP1D1 construct. As evident, the dark MSP1D1 shows negligible fluorescence contribution. This justifies the use of this construct for all our nanoDSF measurements.

# **Electrostatic mapping of CYP2J2 structure**


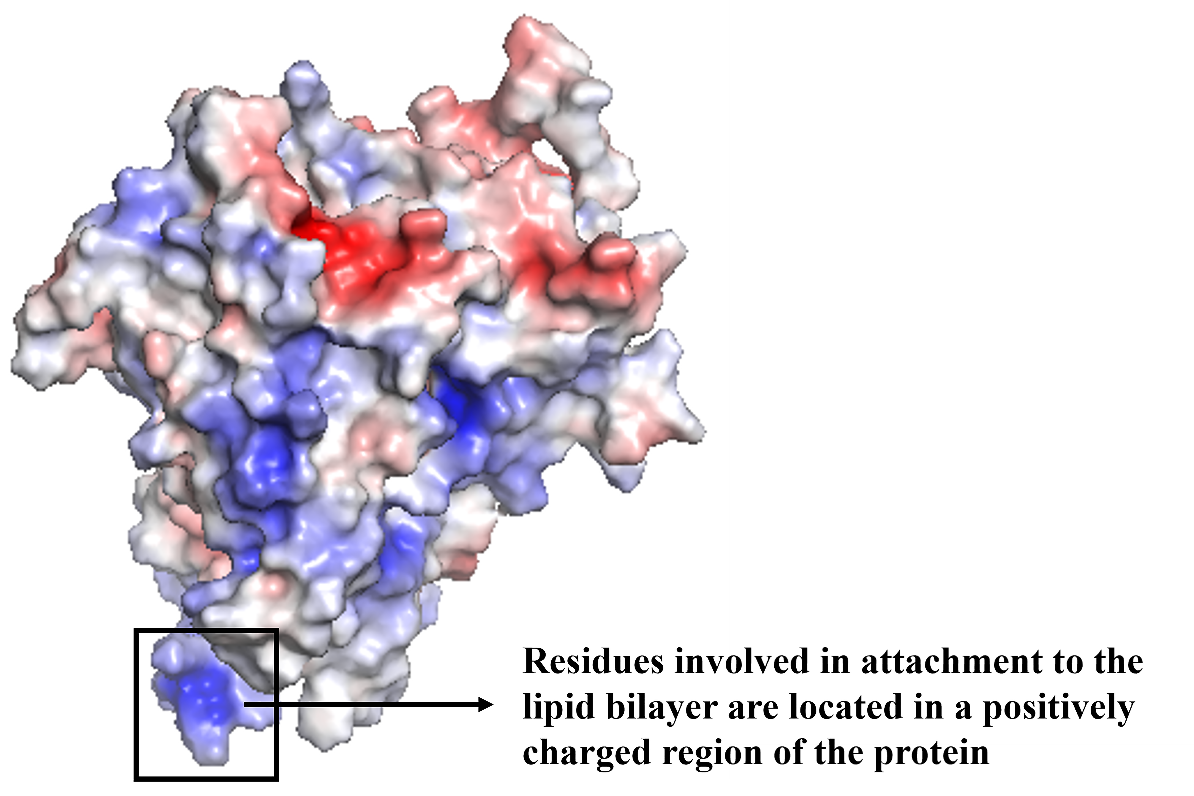


**Figure S3.** Surface projection of electrostatic mapping of CYP 2J2 showing the regions with positive electrostatic potential (Blue) and negative electrostatic potential (Red). Residues involved in binding with the lipid bilayer are located in a positively charged region of the protein. This points to possible electrostatic interactions between the protein and the negatively charged lipids in the membrane leading to more insertion depth of CYP2J2 in the lipid bilayer with POPS lipids.

# **Control experiments for Nano-DSF**


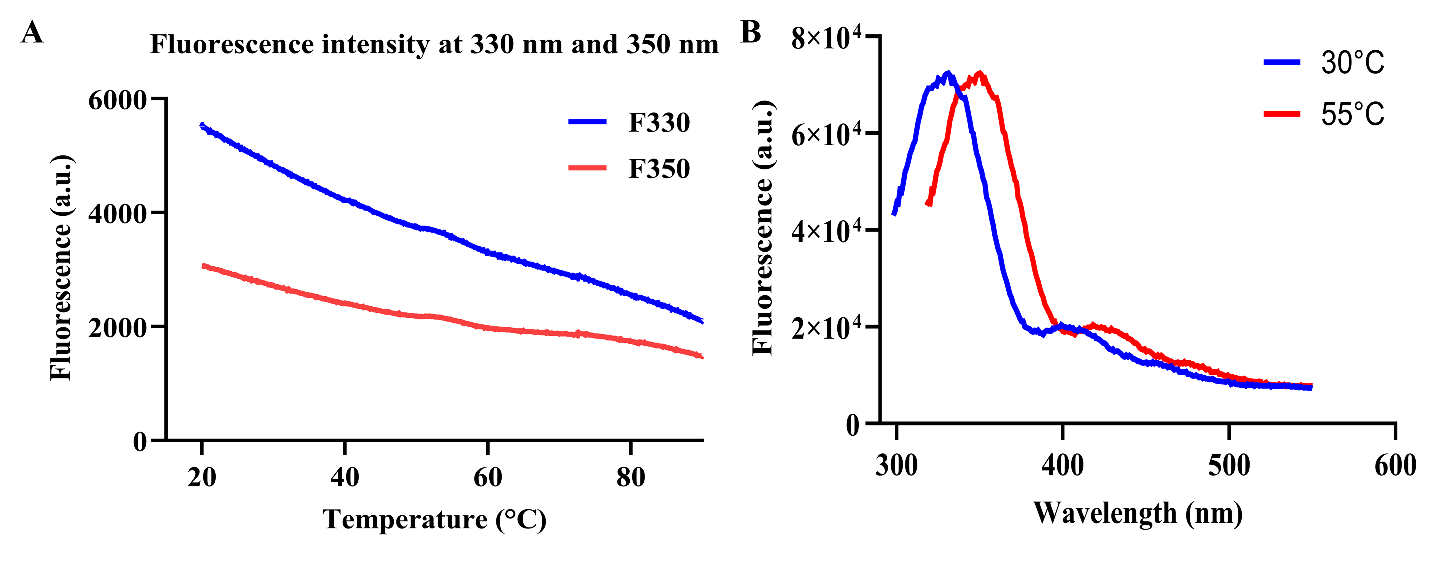


**Figure S4.** (A) Fluorescence intensity of CYP2J2 ND (100% POPC) at 330 and 350 nm with increasing temperature showing a gradual decrease in the intensity due to thermal quenching and (B) Emission spectrum of CYP2J2 ND (100% POPC) at 30**°**C and 55**°**C showing the shift in emission peak wavelength from 330 nm to 350 nm.

# **Thermal unfolding of CYP2J2 ND using UV-Visible Spectroscopy**


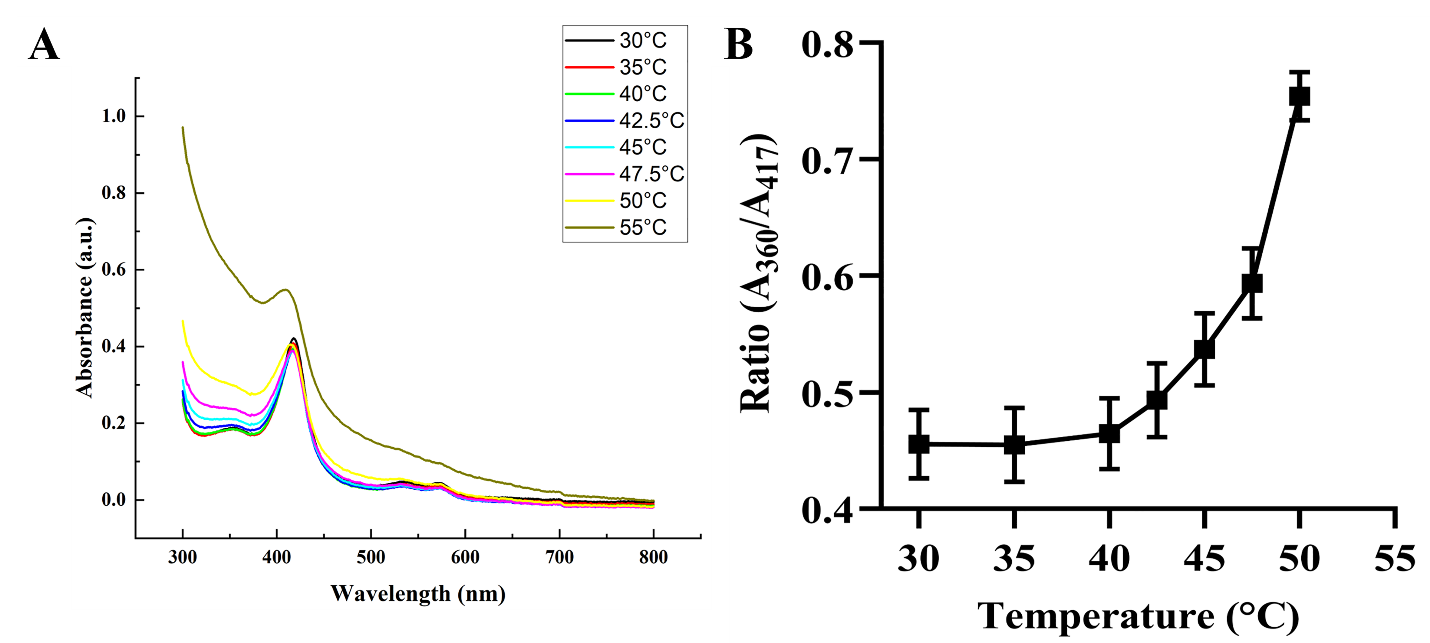


**Figure S5.** (A) Raw spectra (300 nm-800 nm) for CYP2J2 ND made with native ER composition incubated at discrete temperatures (30**°**C to 55**°**C) for 10 minutes; (B) Ratio of absorbances at 360 nm and 417 nm (A_360_/A_417_) plotted against temperature show increasing heme perturbation as a consequence of thermal denaturation. Average of triplicates is plotted with SEM as error bars.
